# Evaluation of Gene Association Methods for Coexpression Network Construction and Biological Knowledge Discovery

**DOI:** 10.1371/journal.pone.0050411

**Published:** 2012-11-30

**Authors:** Sapna Kumari, Jeff Nie, Huann-Sheng Chen, Hao Ma, Ron Stewart, Xiang Li, Meng-Zhu Lu, William M. Taylor, Hairong Wei

**Affiliations:** 1 Department of Mathematical Sciences, Michigan Technological University, Houghton, Michigan, United States of America; 2 Morgridge Institute for Research, Madison, Wisconsin, United States of America; 3 Statistical Methodology and Applications Branch, Division of Cancer Control and Population Sciences, National Cancer Institute, National Institutes of Health, Bethesda, Maryland, United States of America; 4 Division of Animal and Nutritional Sciences, West Virginia University, Morgantown, West Virginia, United States of America; 5 Department of Computer Science, Michigan Technological University, Houghton, Michigan, United States of America; 6 State Key Laboratory of Tree Genetics and Breeding, Research Institute of Forestry, Chinese Academy of Forestry, Beijing, P.R. China; 7 Department of Computer Science, University of Wisconsin, Madison, Wisconsin, United States of America; 8 Biotechnology Research Center, Michigan Technological University, Houghton, Michigan, United States of America; 9 School of Forest Resources and Environmental Science, Michigan Technological University, Houghton, Michigan, United States of America; University of California, Los Angeles, United States of America

## Abstract

**Background:**

Constructing coexpression networks and performing network analysis using large-scale gene expression data sets is an effective way to uncover new biological knowledge; however, the methods used for gene association in constructing these coexpression networks have not been thoroughly evaluated. Since different methods lead to structurally different coexpression networks and provide different information, selecting the optimal gene association method is critical.

**Methods and Results:**

In this study, we compared eight gene association methods – Spearman rank correlation, Weighted Rank Correlation, Kendall, Hoeffding's D measure, Theil-Sen, Rank Theil-Sen, Distance Covariance, and Pearson – and focused on their true knowledge discovery rates in associating pathway genes and construction coordination networks of regulatory genes. We also examined the behaviors of different methods to microarray data with different properties, and whether the biological processes affect the efficiency of different methods.

**Conclusions:**

We found that the Spearman, Hoeffding and Kendall methods are effective in identifying coexpressed pathway genes, whereas the Theil-sen, Rank Theil-Sen, Spearman, and Weighted Rank methods perform well in identifying coordinated transcription factors that control the same biological processes and traits. Surprisingly, the widely used Pearson method is generally less efficient, and so is the Distance Covariance method that can find gene pairs of multiple relationships. Some analyses we did clearly show Pearson and Distance Covariance methods have distinct behaviors as compared to all other six methods. The efficiencies of different methods vary with the data properties to some degree and are largely contingent upon the biological processes, which necessitates the pre-analysis to identify the best performing method for gene association and coexpression network construction.

## Introduction

The use of gene expression data to construct coexpression networks and perform network decomposition [1]–[3] and network analysis [4]–[6] has proven very useful in biological study. However, which methods are more efficient in performing coexpression analysis and constructing coexpression networks has not yet been reported. Such an evaluation is challenging because (1) there is inadequate gene expression data from a specific tissue or cell type over a development stage, or under a specific treatment or condition; (2) genes explicitly involved in a developmental or a biological process are often unclear in higher plants and animals; and (3) we have limited prior knowledge (e.g. positive and negative genes) for comparing the efficiency of different gene association methods in discovering true functionally associated genes. However, since biological data and knowledge are now being accumulated at an unprecedented rate, it is possible to explore the efficiency of gene association methods for constructing biologically meaningful co-expression networks and knowledge discovery in high plants and mammals.

Selecting the best gene association methods for coexpression network construction is important because the methods that can identify genes with true concordance often determine the types and amount of knowledge we can gain from coexpression analysis. Since the genes involved in different activities or biological processes often behave differently and exhibit variable concordance, identification of the best-performing methods is often challenging. For instance, genes involved in different biological processes show discrepancies in response time and coordination strength [2], [7], [8]. In addition, genome-wide studies have shown that gene expression data is intrinsically noisy [9]–[11]. Here noise is defined as unwanted signals from microarray hybridization (technical noise) and stochastic variation arising from interaction of a number of molecules or genes [12]. Noisy gene expression data demands robust methods for biological pattern recognition and true knowledge discovery. Stochastic variation in gene expression can arise simply from a transcription process in which a few dozen or even two hundred general and specific transcriptional factors are assembled into a complex transcriptional machinery where they interact and generate variation in gene expression data even under the same conditions. In this regard, transcriptional machinery in the nuclei is the key convergence point through which a vast array of information from cellular signaling cascades. An early study showed that transcription noise is partly due to variability in upstream signaling [13]. In addition, transcription for a particular gene can occur in bursts and can fluctuate, sometimes (but not always) in synchrony with biological processes such as the cell cycle [14], somitogenesis [15] and transitions between promoter states [13]. As a result, attempting to conclude which gene association method is the best for all purposes or all data types is unrealistic. It is more meaningful to evaluate existing methods for various biological subjects or conditions, and learn their general statistical power in conjunction with their biological context.

In this study, we evaluated eight gene association methods, including Pearson, Spearman rank correlation [1], Hoeffding's D measure [16], Theil-Sen [17], [18], Rank Theil-Sen, Distance Variance [19], Kendall correlation [20] and Weighted Rank [21], to associate pathway genes and regulatory genes. Pearson has been widely used in most coexpression analyses [22]–[24]. Simple linear regression [2], [3], which yields the same order of gene rankings as Pearson, is not included in this study. We used eight methods to associate the pathway genes using the 108 *Arabidopsis* data sets (chips) of Affymetrix ATH1 platform. The dominant biological processes in *Arabidopsis* data sets are stress response, and growth-related processes. The goal for pathway analysis is to examine which methods can associate more genes within the same pathway. The other evaluation we performed is to examine which alternative methods can associate those transcription factors (TFs) that are known to involve or control a biological process coordinately. To achieve this, we took advantage of an existing tool, TF-Cluster, we have recently developed [1]. The TF-Cluster can be used to construct a special coordination network of all TFs, and then decompose it to individual TF sets, each of which contains coordinated TFs controlling a biological process or trait. Details of the method were shown in our previous publication [1]. In original software package, we used Spearman method to associate all TFs to construct a coordination network of all TFs, and in this study, we integrated other seven gene association methods. In addition to 108 *Arabidopsis* data sets (chips), we also used 189 microarray data sets (chips) from human stem cells for associating TFs. Human microarray data sets were collected from multiple experiments in which human embryonic stem cells were treated with different reagents that triggered multiple types of differentiation. In human data, the thriving biological themes are pluripotency maintenance and differentiation. We tried above-mentioned eight methods and found that the Spearman, Kendall, and Hoeffding methods are more efficient than other methods for pathway gene association, whereas Theil-Sen and Rank Theil-Sen perform very well for TFs coordination network. Generally speaking, Distance Covariance and Pearson are less proficient.

## Results

### Evaluation of eight different gene association methods by pathway analysis: top genes

**Figure 1:**
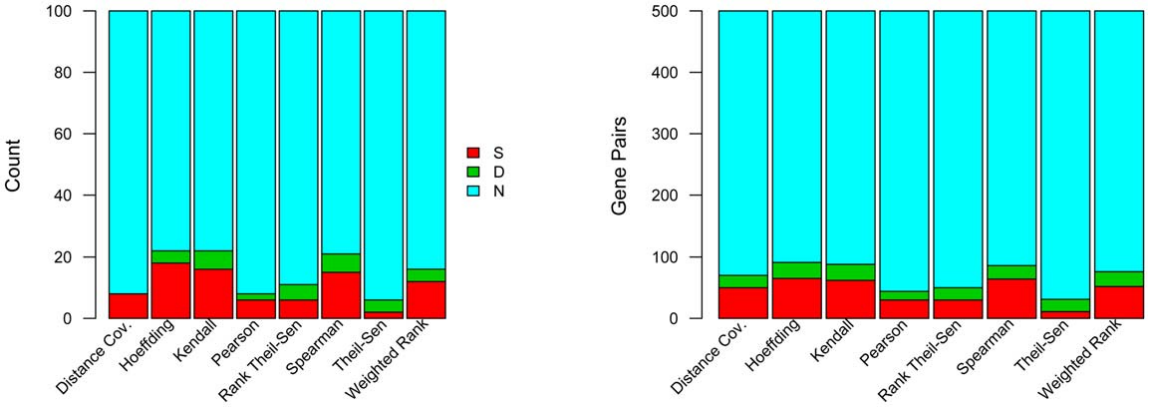
Efficiency of eight methods in associating pathway genes. Relative proportions of coexpressed gene pairs are within the same pathways (S); different pathways (D); and none of existing known pathways (N) in the top 100 (left panel) and 500 (right panel) pairs resulting from the correlation analysis. 576 genes in 30 pathways were analyzed against all genes in the genome.

Genes in the same biological pathway are more likely to be coordinated or co-expressed in order to ensure the co-occurrence of an array of biochemical reactions in response to an internal or external stimulus. This has been shown by previous analysis on transcriptional coordination of pathway genes in *Arabidopsis* [2]. To reduce the computational time, we chose 576 genes in 30 *Arabidopsis* metabolic pathways (Table S1) that are mainly involved in stress response, metabolic processes and wood formation-related processes. We performed pair-wise analysis between each of these pathway genes and all other genes in *Arabidopsis* genome using all eight gene association methods. The output for each method was sorted by p-values in ascending order. We then examined how many genes in the top 100, and 500 pairs, were within the same pathways or in different pathways. The counts reflect the efficiency of different methods in associating the functionally associated pathway genes. The final results were shown in Figure 1. Our findings from this analysis include: (1) Hoeffding, Kendall, and Spearman methods have equivalent performance, whereas Weighted Rank correlation method has an intermediate performance. All the rest have relatively poor performance and should be avoided when co-expression analysis is applied to associating genes involved in metabolic pathways. (2) All methods except Theil-Sen identified more gene pairs containing genes within the same pathways (red bar) than in different pathways (green bar). This indicates that genes within the same pathway have high concordance compared to genes within different pathways. Some methods began to identify more pairs of different pathways only when we examined the top 3,000 pairs or more. (3) In this circumstance, the Distance Covariance method does not identify any pair of genes in different pathways when the top 100 pairs were selected, whereas Pearson identified the least number of pairs containing genes of different pathways when the top 500 pairs were examined, suggesting that it is less robust, and may not identify some kinds of pathway gene coordination that could be identified by other methods.

To further examine the sensitivity, specificity and predicted accuracy of eight gene association methods for pathway gene association, we made some assumptions. We sorted all genes coexpressed to 576 pathway genes in ascending order with the most tightly coexpressed gene pairs located at the top. For each gene list resulting from one of the eight methods, we cut off the top100 pairs, and assumed that pairs that are of the same pathway are true positives (TP), and all pairs that are not in the same pathway and non-pathway genes are false positive (FP). To obtain true negatives (TN) and false negatives (FN), we cut off three slices from each sorted paired gene list. Slice 1 contains genes from top 101 to 1000 pairs, Slice 2 contains 900 gene pairs from the mid of gene list, and Slice 3 contains 900 gene pairs from the bottom of the sorted gene list. We then defined any pairs of genes that are of the same pathway in a given slice as false negatives (FN), and any pairs of genes that are not within the same pathway in the same slice as true positives (TN). Since each of these slices contains 900 genes, we divided the gene numbers by 9 before we compared the numbers we obtained from the top 100 gene pairs. We then calculated sensitivity, specificity, and predicted accuracy when the top 100 genes were compared to any of these three slices. The results are shown in Table S2. We also calculated the sensitivity, specificity, and predicted accuracy of genes belonging to different pathways. In this case, gene pairs within the top 100 genes that are of different pathways were defined as TP, and genes of the same pathways or non-pathways were defined as FP. TN and FN were obtained from three slices in the same principle. We also applied above-mentioned analyses to the top 500 gene pairs and three slices, each of which contain 500 genes from the top 501∼1000s gene pairs, and the middle and the bottom of each gene list. The FN, FP, TP, and FP obtained from the top 500 genes and one of these three slices was used directly to calculate the sensitivity, specificity and predicted accuracy. The results shown in Table S2 indicate that Hoeffding, Kendall and Spearman and Weighted rank generally have higher sensitivity, specificity, and predictive accuracy, reinforcing the conclusion drawn from Figure 1 that Hoeffding, Kendall, and Spearman methods have equivalent performance, and that Weighted Rank Correlation is the next best method for pathway analysis. See Table S2 for more detail.

### Evaluation of eight different gene association methods by pathway analysis: p-value

Although examination of a certain number of top genes is rational in biological analysis, we were also interested in learning the efficiency of each method if the output of pathway analysis of each method were cut off by a threshold p-value. Since the resultant p-values from different methods can be in different orders of magnitudes, we obtained substantial number of significant gene pairs for some methods and a small number of significant gene pairs for other methods when implementing the same cut-off p-value threshold (e.g. p value <0.05) on different methods, making it difficult to compare the efficiency of different methods (see Table 1 below). However, when we had a series of different cut-off p values, it was generally true that rates of within-pathway gene pairs resulting from Kendall, Spearman, and Weighted appeared to be higher than Pearson, Theil-Sen and Rank Theil-Sen. In addition, it was obvious that Spearman, Kendall, and Pearson methods have a very wider range of p values than any other methods. Hoeffding and Distance Covariance method have a smaller p-value range (between 1×10^−10^ and 1×10^−5^). This sometimes can make it difficult to obtain a proper number of genes with one threshold p-value.

**Table 1:** The percentage of gene pairs in the same pathway when p-value thresholds ranging from 1.0×10^−35^ to 1.0×10^−5^ were applied to cut off correlated lists of gene pairs resulting from eight methods.

Methods\P value	1.0E-35	1.0E-25	1.0E-15	1.0E-05	0.05
**Kendall**	2.51% (25,485)	2.51% (25,499)	1.76% (41,749)	0.25% (1,364,076)	0.15% (5,272,820)
**Spearman**	12.87% (404)	5.31% (1,611)	1.13% (87,116)	0.24% (1,472,610)	0.15% (5,312,831)
**Rank Theil-Sen**	/	/	0.30% (886,264)	0.15% (5,112,691)	0.13% (8,032,269)
**Hoeffding**	/	/	/	/	0.14% (6,927,518)
**Weighted Rank**	/	/	/	0.26% (1,279,295)	0.15% (5,287,220)
**Theil-Sen**	/	/	0.36% (636,501)	0.16% (4,695,948)	0.13% (7,847,207)
**Pearson**	1.02% (17,701)	1.09% (49,630)	0.71% (200,325)	0.24% (1,725,471)	0.15% (5,486,544)
**Distance Covariance**	/	/	/	/	0.16% (4,264,095)

(The numbers shown in parentheses are gene pairs in cut-off lists by p values).

### Coexpression connectivity of genes within the same pathway and different pathways

**Figure 2:**
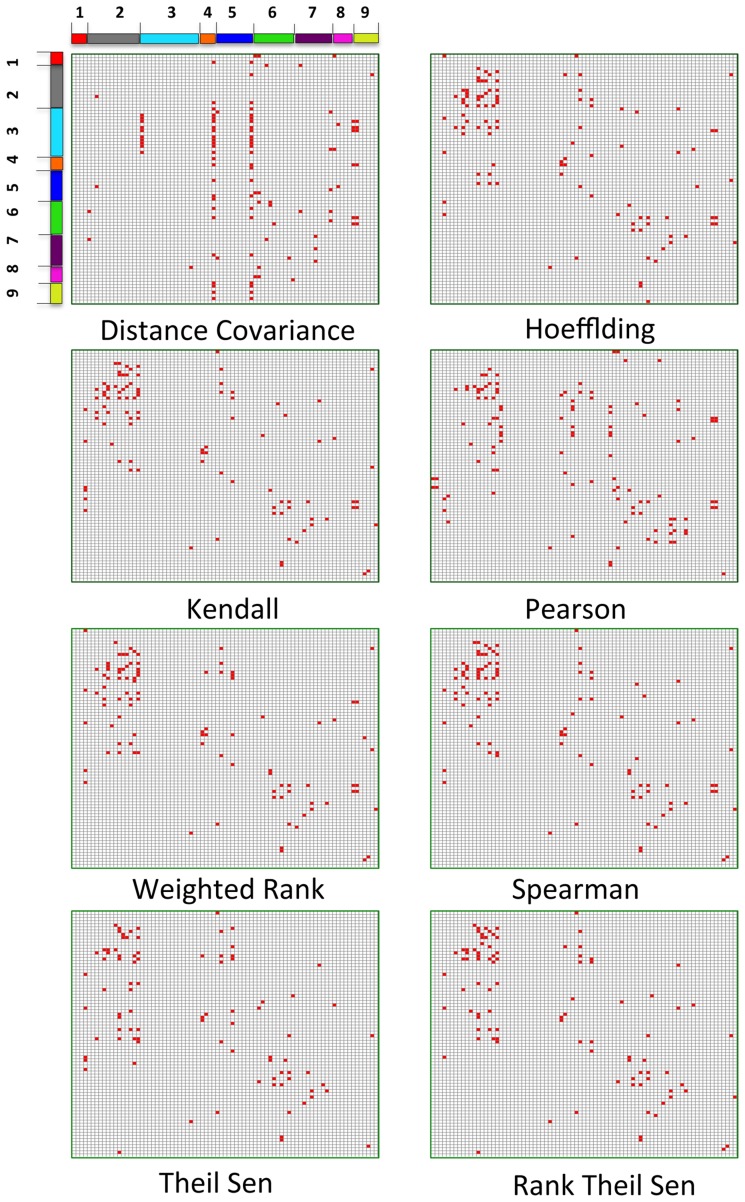
Common and distinct coexpression patterns recognized by eight methods. Within and across-pathway gene coexpression connectivity patterns when eight gene associated methods were used. Coexpression relationships are viewed as a heatmap between any two of 9 selected pathways that represent stress response and primary metabolism, and wood formation, which are labeled from 1 to 9. 1) abscisic acid biosynthesis (4 genes), 2) aerobic respiration (14 genes), 3) gluconeogenesis (16 gene), 4) glycolysis IV (plant cytosol) (4 genes), 5) glyoxylate cycle (10 genes), 6) IAA biosynthesis I (10 genes), 7) Phenylpropanoid biosynthesis (10 gene), 8) UDP-galactose biosynthesis (5 genes), 9) UDP-D-xylose biosynthesis (7 genes). For each of 81 genes in these 9 pathways, we obtained the top 100 most coexpressed genes to it by performing a genome-wide coexpression analysis in which each of these 81 genes was paired with all other genes in the genome. We plotted all those pairs, in which both genes are one of these 81 genes in any of these 9 pathways.

To show the discrepancy of coexpression patterns of genes within the same pathway and different pathways resulting from eight genes association methods, we plotted a window that displayed 81 genes in 9 pathways of our interest or being stress-related (Figure 2). Coexpression patterns identified by Hoeffding, Kendall, Weighted Rank, Spearman, Theil-Sen and Rank Theil-Sen were similar to each other though it appears that coexpression patterns identified by Theil-Sen and Rank Theil-Sen are relatively more cognate. The patterns identified by Distance Covariance and Pearson were noticeably different from all others though Pearson's was closer to those identified by Hoeffding, Kendall, Weighted Rank, and Spearman methods. The results shown in Figure 2 suggest that the efficiency of these eight methods varies with the pathways. For example, Distance Covariance, Hoeffding, Kendall, Pearson, Spearman, Weighted Rank, Theil-Sen, and Rank Theil-Sen identified 1, 29, 29, 19, 32, 25, 21, and 28 connections within aerobic respiration pathway, respectively, whereas the eight methods in the same order identified 2, 5, 4, 11, 5, 5, 4 and 4 correlation relationships in the phenylpropanoid biosynthesis pathway.

### Evaluation of eight different gene association methods by network construction followed by decomposition

We developed a novel approach for identifying these regulatory genes that control a trait or a biological process by building a conceptually new coordination network of all transcription factors (TFs) and then decomposing it into multiple clusters using a heuristic algorithm called Triple Link we recently developed [1]. We demonstrated that each cluster contains a set of regulatory genes controlling a trait or a biological process, which has enormous practical implications by providing a means to increase yield and quality in an agricultural context. The details of how to build the coordination network and how to decompose the coordination network were shown in our previous publication [1]. Briefly speaking, two TFs, “A” and “B”, are connected in the coordination network only if the top n most closely coexpressed genes to A and the top n most closely coexpressed genes to B have more than k genes in common (k<n, k and n are dynamic but usually n = 100, k = 30). We then store k in a symmetric matrix where both dimensions are all TFs from a specific genome. If converted to a graph, such a matrix actually represents a coordination network of all TFs. Triple-link works as follows: it first searches all connected node pairs (genes) in the co-expression network, and identifies the pair with highest k. This pair is then used as a primer for growing into a TF cluster as follows: a third TF is joined in if it has a significant connectivity to each of this pairs (more than the average of the matrix plus at least one standard deviation), and thereafter, all TFs that are subsequently joined in need to have at least three significant connectivities to the TFs already in the cluster. The cluster stops growing until there are no more nodes (TFs) meeting the required connectivities. A TF cluster is then produced. All TFs in this cluster are removed from the TF matrix, and they do not participate in the next round of decomposition. This process is repeatedly executed until all TFs in matrix are classified into multiple clusters. We then demonstrated that many of the resulting TF clusters contain functionally coordinated TFs that, based on existing literature, regulate a biological process/trait of interest.

In this study, we used the previously identified TFs in the clusters shown earlier [1] as positive genes (also provided in Table S3). TFs in each of these clusters are supported by existing literature to be functionally associated and to collectively control a biological process or trait [1]. We attempted to see if each new method could associate them together into one cluster. We built 16 coordination networks of all transcription factors, (1,640 TFs from *Arabidopsis*; 2,180 TFs from human), using eight gene association methods and two compendium data sets (see Methods and Materials) following the procedure described [1]. We then used the Triple-Link algorithm to decompose these 16 TF coordination networks to obtain TF clusters; each is postulated to control a trait or a biological process. We then examined the presence of these positive genes in the top 25 clusters. In addition to the number of positive genes, a cluster number is the other indicator that can tell how efficient a method is. If a cluster is recognized by a method with a smaller cluster number (e.g. Cluster 5 is smaller than Cluster 15), this indicates that the method can associate the TFs in this cluster with higher strength. As a result, it is picked up by Triple-Link at earlier stage of decomposition. The resultant outcomes from *Arabidopsis* and human are described below.

### *Arabidopsis* data: Which gene association methods can identify more positive genes and a higher percentage of positive genes?

**Figure 3:**
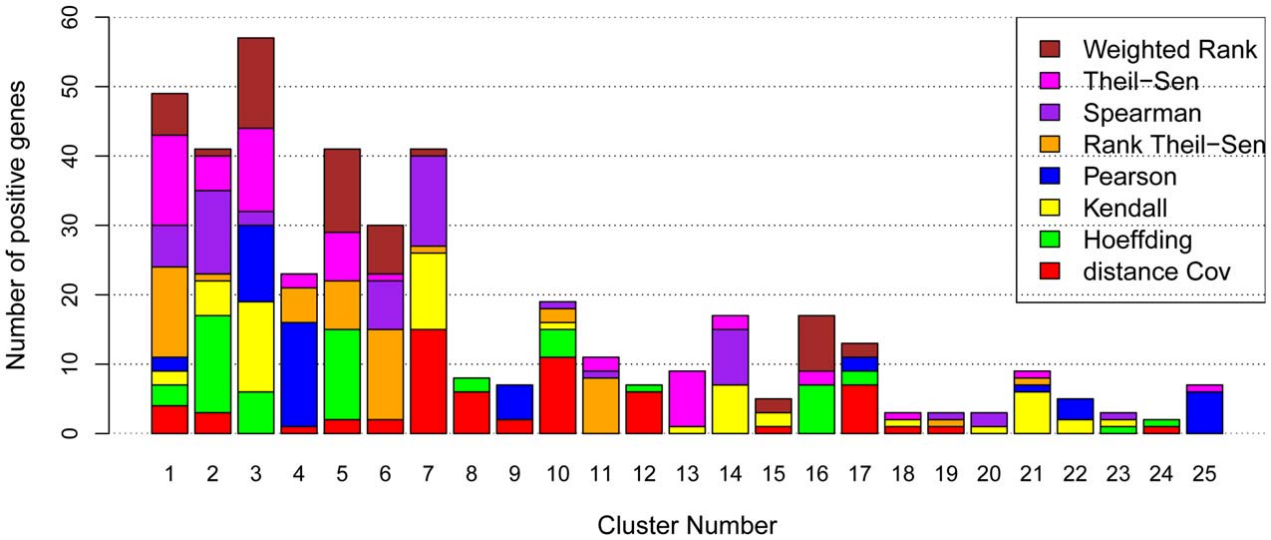
Efficiency of eight methods in associating regulatory genes in *Arabidopsis*. The numbers of positive TFs in top 25 clusters identified by TF-Cluster [1] when eight gene association methods were used to construct the coexpression network of all TFs (1640 in Arabidopsis ATH1 platform) for network decomposition to recognize the positive TF clusters regulating different biological processes. These TFs were from Nie at al Table 2 in [1] where literature evidence that support them to be positive genes are shown.

We first performed genome-wide co-expression analysis using eight gene association methods. In this analysis, each of 1,640 TFs was paired with all other genes (including each of the other 1639 TFs) in the *Arabidopsis* genome, and computed using eight gene association methods on Linux cluster containing 2500 nodes through Condor, a large collection of distributive computing resources across University of Wisconsin campus. The coordination of two TFs was measured by the number of common genes present in the top 100 most coexpressed genes to each of these two TFs. The resulting coordination networks of all 1,640 TFs from eight gene association methods (represented by eight matrices) were then decomposed with Triple-Link algorithm as described earlier [1] to identify the TF sets, each containing a group of TFs that collectively regulate a trait. After network construction and decomposition, we obtained many clusters that contained positive TFs shown in Table 2 of in the original publication [1] for all of eight gene association methods. These positive TF genes are implicated to control several biological traits in *Arabidopsis* roots that include root cap development, root hair development, root vascular development, root cell cycle, and drought response to abscisic acid (ABA) (Table S3). The different numbers of positive genes in top clusters recognized by TF-Cluster due to use of eight gene association methods are shown in Figure 3. For all the eight methods, the positive TFs are present in the top seven TF clusters. This is because TF-Cluster was designed in such a way that the TF set with more tightly coordinated TFs clusters is provided earlier. Combining all top seven clusters, the methods perform in the following order in respect to the number of the positive genes identified: Theil-Sen (40 positive TFs)  = Rank Theil-Sen (40)  =  Spearman (40 Positive TFs)  =  Weighted Rank (40 positive TFs) > Hoeffding (36) > Kendall (31) > Pearson (28 positive TFs) > Distance Covariance (27 positive TFs). The numbers in the parentheses are those positive TFs identified by each method. We ranked Theil-Sen and Rank Theil-Sen before Weighted Rank and Spearman methods because they identified larger clusters during the earlier stage of decomposition.

**Table 2:** The performance order of eight gene association methods in first seven clusters.

Cluster	Order
1	Weight Rank (75%) > Spearman (67%) > Rank Theil-Sen (61%) > Theil-Sen (48%).
2	Kendall (71%) > Hoeffding (58%) > Spearman (56%) > Theil-Sen (45%)
3	Hoeffd (85%) > Weight Rank (64%) > Theil-Sen (65%) > Kendall (58%)
4	Rank Theil-Sen (56%)
5	Rank Theil-Sen (80%) > Hoeffd (78%) > Weight Rank (62%) > Theil-Sen (62%)
6	Rank Theil-Sen (73%) > Weight Rank (62%) > Spearman (57%)
7	Spearman (82%)

**Figure 4:**
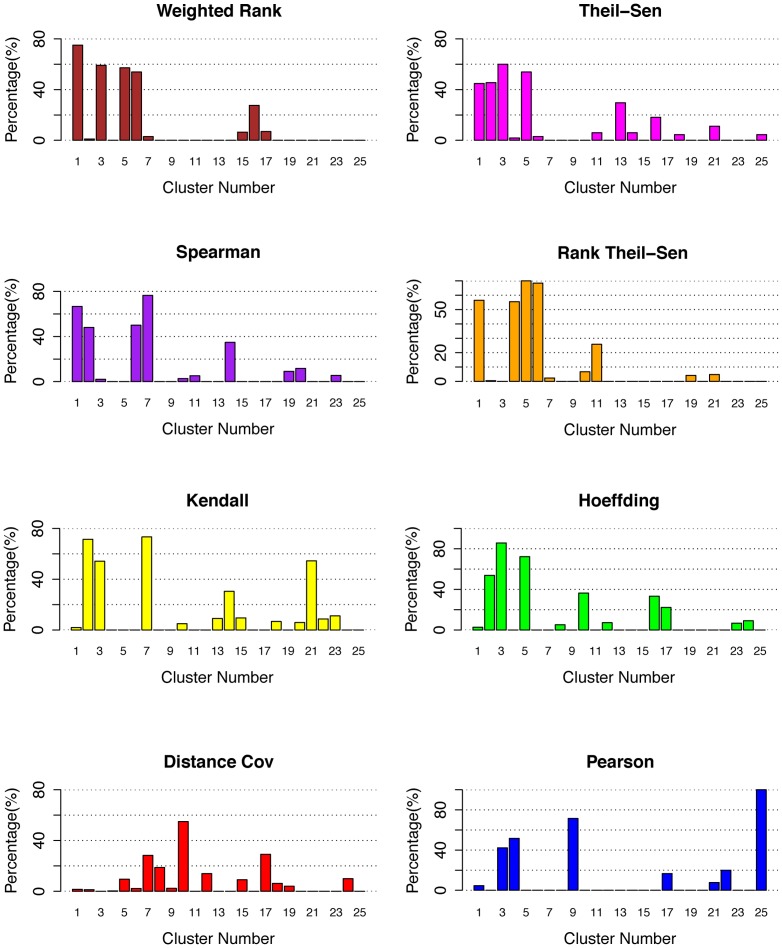
Efficiency of eight methods in associating regulatory genes in *Arabidopsis*. The percentage of positive genes in 25 top clusters identified by network as described early [1] when eight gene association methods were applied to *Arabidopsis* microarray data sets of 108 samples.

The use of total number of positive TFs identified by different methods to evaluate eight gene association methods can be biased because the size of each cluster was not taken into account. For this reason, we also investigated the percentage of positive genes in each cluster, and the results were shown in Figure 4. From these results, we can observe that most methods including Weighted Rank, Rank Theil-Sen, Spearman, Theil-Sen, and Hoeffding could generate highly ranked clusters with high percentage of positive genes (Table S4). The fact that most clusters contain functionally cohesive TFs suggests that coordinated TFs controlling the same traits were successfully associated and led to discovery of novel knowledge. To compare these methods more precisely, we listed the discovered positive gene rates in Table 2. For accuracy, the five methods ranked as Hoeffding (74%), Kendall (70%), Rank Theil-Sen (68%), Spearman (65%), Weighted Rank (65%), and Theil-Sen (55%). Although Hoeffding and Kendall had higher positive gene rates, there were only three clusters that had more than 40% positive genes while other methods, Spearman, Rank Theil-Sen, and Weighted Rank and Theil-Sen, had four clusters with more than 40% positive genes. What was missing was a cluster of TFs controlling root cell cycle. We examined the genes and found Kendall had that cluster in Cluster 21 while Hoeffding split it into Cluster 10 and Cluster 17 (Table S4). Considering the positive gene numbers and positive gene rate in each cluster, we conclude that Spearman, Weighted Rank, Theil-Sen, and Rank Theil-Sen have a more robust and powerful performance.

### Human data: Which gene association methods can identify more positive genes and a higher percentage of positive genes?

**Figure 5:**
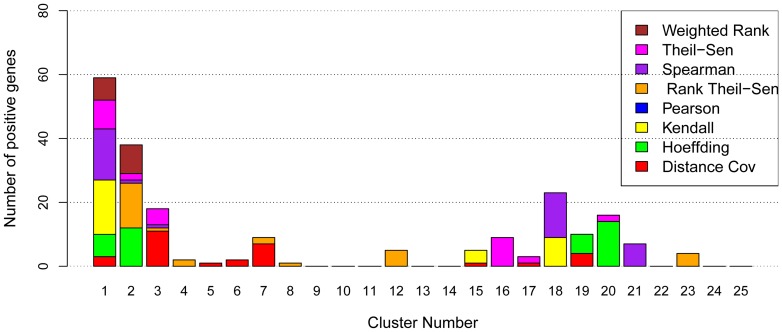
Efficiency of eight methods in associating regulatory genes in human. The number of positive regulators in the top 25 TF clusters identified by network construction and decomposition using TF-Cluster when eight gene association methods were applied to the human microarray compendium data set containing 189 chips.

We investigated the efficiency of eight gene association methods for identifying TFs controlling several biological processes in human stem cells undergoing differentiation. We first performed genome-wide co-expression analysis in which each of 2,180 TFs was paired with all genes in the human genome (including each of 2,179 other TFs) and computed using eight gene association methods on a NIH Linux Cluster (http://biowulf.nih.gov/). We then cut off the top 100 most coexpressed genes to each TF and built eight coordination networks, which were subsequently decomposed with Triple-Link Algorithm [1] to identify the TF groups containing positive TFs. The positive TFs are those listed in Table 1 of our earlier publication (Nie, Stewart et al. 2011) and which is also shown in Table S3. After network construction and decomposition, we searched these positive TFs in top 25 clusters (shown in Figure 5). Spearman is the original method integrated in the TF-Cluster for gene association, and it identified 16 positive genes in Cluster 1, but Kendall identified 17 positive in Cluster 1. In the same cluster, the Theil-Sen, Weighted Rank and Hoeffding methods identified 9, 7, and 7 positive genes respectively. In cluster 2, Rank Theil-Sen and Hoeffding and Weight Rank identified 14, 12, and 9 positive genes respectively. In Cluster 3, Distance Covariance, and Theil-Sen identified 11, and 5 positive genes respectively. Given such results, although Theil-Sen and Weight Rank and Hoeffding identified a number of positives in Cluster 1, 2 and 3, the genes involved in pluripotency are separated into two clusters. Considering the number of positive genes in each cluster and occurrence of breakdown of functional clusters, we ranked the eight methods in this order, Kendall > Spearman > Theil-Sen > Weighted Rank > Hoeffding> Rank Theil-Sen. All other methods performed poorly.

**Figure 6:**
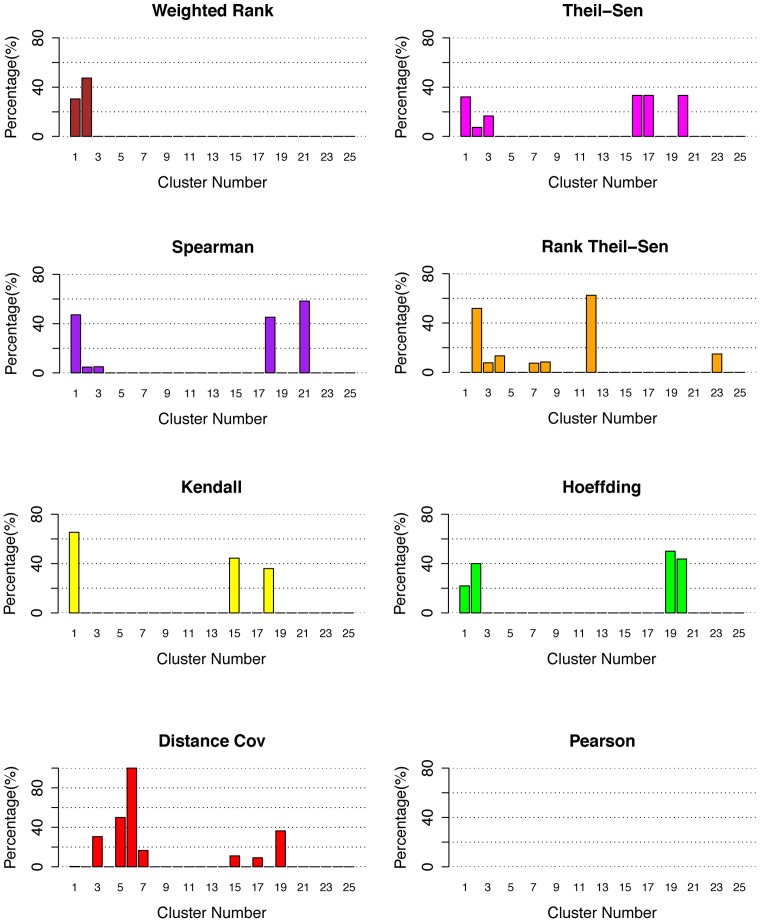
Efficiency of eight methods in associating regulatory genes in *human*. The percentage of positive genes in the 25 top clusters derived from human data from stem cells underwent differentiation by coexpression network construction and decomposition as described [1] when eight gene association methods were used.

Similar to analyses performed to *Arabidopsis* data, examining the number of positive TFs present in top clusters derived from TF-Cluster pipeline by different methods is inadequate. For this reason, we investigated the percentage of positive genes (Figure 6). For the three clusters (Cluster 1, 18, 21 shown in Figure 6) obtained by Spearman that contain TFs controlling pluripotency, multiple directional differentiation and neural development, respectively, Hoeffding split the Cluster 1 into Cluster 1 and 2. Kendall split Cluster 21 into multiple clusters (not shown in Figure 6 because they have a larger cluster number >25) (Table S4). Weighted Rank also split the Cluster 1 from the Spearman method into Cluster 1, and 2, and Cluster 18 and 21 into many small clusters with a cluster number larger than 25. Theil-Sen split the Cluster 1 of Spearman method into Cluster 1, and 3 (Table S4), Cluster 18 into 2 and 16, and Cluster 21 into many small clusters (not shown due to high cluster numbers >25). Rank Theil-Sen split Cluster 18 of Spearman method into Cluster 4, 23 and multiple small clusters (Table S4). In consideration of positive gene rates in different clusters, we think Kendall, Hoeffding, and Rank Theil-Sen can be a competitive method for Spearman.

### Is the efficiency of eight gene association methods contingent on the gene functions?

**Figure 7:**
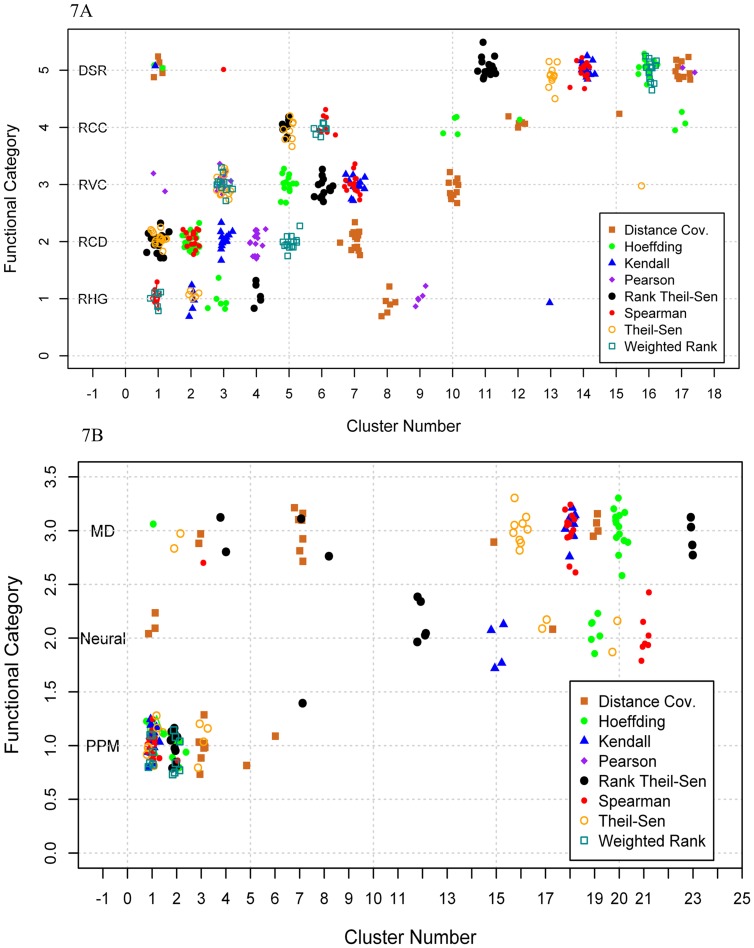
Performance of eight gene association methods is contingent on biological processes. 7A, Clusters verse biological events in Arabidopsis: RHG: root hair growth, RCD: root cap growth, RVC: root vascular development, RCC: root cell cycle, DSR: drought response to ABA. 7B. Clusters verse biological events in human: PLM: pluripotency maintenance, ND: neural development; MDD: multi-direction differentiation.

To investigate if the performance of the eight gene association methods varied with the biological processes thriving in the data, we classified the 418 positive genes present in the top 17 clusters of *Arabidopsis* shown in Figure 3 into 5 functional categories: (1) RHG: root hair growth, (2) RCD: root cap development, (3) RVD: root vascular development, (4) RCC: root cell cycle, and (5) DSR: drought stress response to ABA, and plotted them in Figure 7A. Each method was evaluated based on the number of positive genes and the rankings of clusters. Since TF-Cluster was designed in such a way that a TF set with more tightly associated TFs is outputted earlier”, a method is considered to have better performance if the derived clusters are highly ranked (with smaller cluster number, or on the left within each row). Based on these rules, it is obvious that the Rank Theil-Sen method has a relatively stable performance for all functional categories, followed by Theil-Sen and Spearman and Kendall methods. The Pearson method had low efficiency for RHG and DSR, and did not capture any genes in RCC. The Distance Covariance method performed poorly for identifying genes in all categories except DSR, whereas the Pearson did not identify any genes in RCC and performed poorly in all categories except RVC. For knowledge discovery, the Spearman method performed slightly better than the Kendall method and the latter did not identify any genes in RCC, but Spearman did. The Rank Theil-Sen, Theil-Sen, Spearman, and Kendall methods identified the genes in the same clusters, suggesting these methods share some common properties.

We also classified 191 positive genes present in the top 23 clusters of human shown in Figure 5 into three functional categories: PPM: pluripotency maintenance; ND: neural development; and MDD: multi-direction differentiation and plotted them into Figure 7B. We found that the efficiency of eight gene association methods varies with the biological processes. No method consistently performed best across all functional categories. For example, Spearman, and Kendall are most efficient methods for PPM category because they are able to associate these TFs controlling PPM together and output them in first cluster. However, their performance in neural and MD categories are less efficient because the clusters were generated in late stage of decomposition, suggesting variable efficiencies when biological processes are altered. In addition, Distance Covariance performed poorly in all five categories in *Arabidopsis*, but could generated positive TF Clusters in an earlier stage for all three categories in human though it split the cluster of the same function into multiple clusters. Based on the results shown in Figure 7B, we can also conclude that the efficiency of eight gene association methods is contingent on the biological processes in human data.

### Relationships between normality and performance of different methods

**Figure 8:**
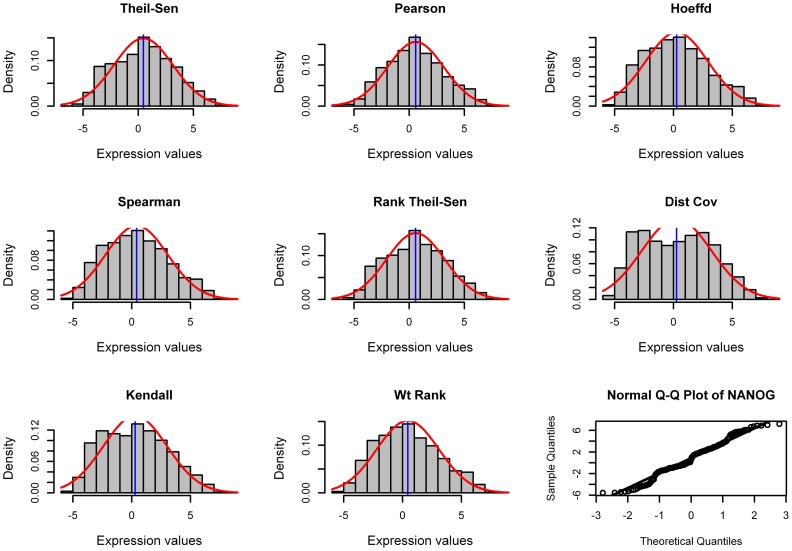
Genes recognized by different methods have different distribution. Distribution of top 100 genes most closely associated with NANOG when eight gene association methods were applied for pairwise analysis. This analysis was done with 189 human microarray data sets as inputs. The approximate normality of NANOG is shown at the right bottom corner by Q-Q plot, in which the points fall on the reference line (solid line at 45°). Additionally, Shapiro-Wilk test shows NANOG has a *W* statistic of 0.98.

To investigate if different methods tend to associate the genes with normal distribution – the values are symmetrically distributed with the majority concentrated around the mean and data follows a bell-shape curve – we chose one gene, NANOG, whose expression values from 189 chips obey an approximate normal distribution. We performed pairwise analysis using eight gene association methods between NANOG and all other genomic genes, and then chose the top 100 most closely correlated genes to NANOG for each method. We then examined the distribution of these top 100 coexpressed genes recognized by each method as a lump, which show if a method tends to associate genes with normal distribution (Figure 8).

The genes associated by the Pearson method tended to have a normal distribution when the gene of interest (e.g, NANOG) had a normal distribution. The Rank Theil-Sen method appeared to associate genes with an approximate normal distribution. Spearman, Hoeffding and Weighted Rank methods captured genes with approximate normal distribution but with bias to the left side. Compared to these three methods, Kendall method identified genes with more bias to the left. The Distance Covariance method identified more genes far from normal than any other methods, indicating it can capture genes with various kinds of relationships. However, we do realize that it is insufficient for us to examine just one gene of interest and also the distribution of the top 100 genes as a whole.

**Figure 9:**
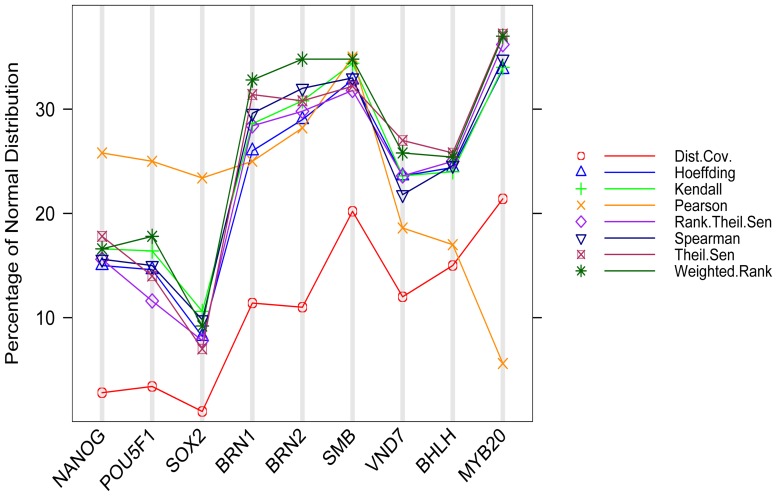
Behavior of eight gene association methods in identifying genes with normality distribution. The percentage of genes with approximately a normal distribution of the top 500 genes most tightly coexpressed to each of 9 selected genes as examined with Shapiro-Wilk test (Significance level p<0.01). Pairwise analysis was performed between each of 9 genes and all genes in the genome, and the results were sorted by p values before top 500 genes were cut-off.

To examine more genes, we chose 9 genes of interest based on some of features as shown in Table 3. For the first three genes known to be the master TFs controlling human stem cell pluripotency [25], any pairs of them have Spearman rho > Pearson r. For the second three genes known to control root cap maturation [26], any pair of them have Spearman rho ≅ Pearson r. For the last three genes known to control secondary cell wall growth [27]–[29], any pair of them have Spearman rho < Pearson r). We showed the percentage of genes with normality distribution in the top 500 genes that are coexpressed to each of these 9 selected genes (Figure 9). The normality is defined as p value <0.01 in Shapiro-Wilk testing. Surprisingly, except Pearson and Distance Covariance methods, all other methods captured approximately the same number of genes with normal distribution but Weighted Rank appeared to capture slightly more genes with a normal distribution. The Distance Covariance method always captured fewer genes with normal distribution in most circumstances. Interestingly, Pearson method captured more normally distributed genes than any other methods when NANOG, POU5F1 or SOX2 were the gene of interest. We found that any pair involving any of these three genes has a Spearman rho that is much larger than and Pearson coefficient r (Table 3). In contrast, the Pearson method captures many fewer genes with a more normal distribution than other methods when VND7, bHLH, or MYB20 were the genes of interest, and any pair of these three genes had a Spearman rho smaller than the Pearson coefficient r (Table 3). Finally, the Pearson method captured the same number of genes with normal distribution when any one of BRN1, BRN2 and SMB were used as the gene of interest, and any pair of these three genes had a Spearman rho approximately the same as Pearson coefficient r.

**Table 3:** Nine genes of interest, each of which was used as one gene in pairwise genome-wide coexpression analyses using eight gene association methods.

Group	Gene	W	Gene	W	Spearman rho	Pearson r
I	POU5F1 (NM_203289)	0.81	NANOG (NM_024865)	0.98	0.77	0.49
I	POU5F1 (NM_203289)	0.81	SOX2 (NM_003106)	0.89	0.72	0.51
I	SOX2 (NM_003106)	0.89	NANOG (NM_024865	0.98	0.73	0.54
II	BRN2 (AT4G10350)	0.87	BRN1 (AT1G33280)	0.89	0.92	0.89
II	SMB (AT1G79580)	0.78	BRN1 (AT1G33280)	0.89	0.68	0.67
II	SMB (AT1G79580)	0.78	BRN2 (AT4G10350)	0.87	0.72	0.74
III	VND7 (AT1G71930)	0.80	MYB20 (AT1G66230)	0.96	0.40	0.68
III	bHLH (AT1G68810)	0.83	MYB20 (AT1G66230)	0.96	0.31	0.49
III	VND7 (AT1G71930)	0.80	bHLH (AT1G68810)	0.83	0.50	0.61

*W* is the statistics of Shapiro-Wilk test.

Given the observations above, it is intriguing to examine how many genes pairs have a Spearman rho approximately the same as Pearson r, as well as how many gene pairs are of normal distribution. Examining gene pairs in this way is important for determining if normality constitutes the basis that lead to some methods to perform better than another, and how the performance of different methods varies when data properties change. To this end, we applied the Pearson and Spearman rank correlation tests to *Arabidopsis* data and then classified all pairs of TF genes into 10 categories (Table 4). Category I has the pairs whose (Spearman rho – Pearson r) >0.1, and gene pairs belonging to this category are only 5.2% (I-1: 1.85% + I-2: 0.05% + I-3: 3.32%) of all gene pairs. Category II has the absolute difference (|Spearman rho – Pearson r|) <0.1, and comprises 84.46% (II-4: 20.73% + II-5: 0.08% + II-6: 0.09% + II-7: 63.56%) of all gene pairs, whereas Category III has (Pearson r – Spearman rho) >0.1, and contains 10.32% (III-8: 0.83% + III-9: 0.14% + III-9: 9.35%) of all gene pairs. The fact that 84.46% gene pairs belong to Category II indicates that most methods, except the Distance Covariance method, do not make much difference even though Weighted Rank, Spearman and Theil-Sen methods tended to capture more normally distributed genes. We applied Shapiro-Wilk test to all genes in different categories with a high stringency (P<0.01). When all gene pairs are concerned, only 16.1% pairs of genes are both normal, and 43.5% pairs have one gene being normal and 40.3% both genes being non-normal (Table 4). In Category II, 41.9% genes have a normal distribution while only 22.9% in Category I and 12.7% genes in Category III have normal distribution. These data support the advantages of using non-parametric methods under all circumstances. However, Category III where (Pearson r – Spearman rho) >0.1 contains highest ratios of genes with non-normality distribution than any of other two categories. We did not find any evidence that the Pearson method favored normal distribution more than a non-parametric method like Spearman method in Category II and III data (Figure 9). In Category III, the number of genes with normal distribution associated by Pearson declined. However, The fact that Pearson achieved higher correlation (r > rho) in Category III supports the notion that Pearson correlation performs well even under circumstances where the data are from non-normal distribution.

**Table 4:** Gene pairs that are classified into 10 types based on Pearson's r and Spearman's rho.

Category		Classifier	No. of GP	P2 (%)	Both genes are normal	One gene is normal	Both genes are non-normal	P2 (%)
**I**	**1**	**rho-r >0.1, r<0, rho <0**	**2343**	1.85%	51	907	1385	21.5%
	**2**	**rho-r >0.1, r<0, rho >0**	**69**	0.05%	1	33	35	25.4%
	**3**	**rho-r >0.1, r>0, rho >0**	**4212**	3.32%	148	1694	2370	23.6%
**II**	**4**	**abs(r-rho) <0.1, r <0, rho <0**	**26293**	20.73%	5310	13660	7323	46.2%
	**5**	**abs(r-rho) <0.1, r <0, rho >0**	**96**	0.08%	16	35	45	34.8%
	**6**	**abs(r-rho) <0.1, r >0, rho <0**	**119**	0.09%	28	64	27	50.4%
	**7**	**abs(r-rho) <0.1, r >0, rho >0**	**80600**	63.56%	14644	35950	30006	40.5%
**III**	**8**	**r-rho >0.1, r<0, rho <0**	**1057**	0.83%	44	564	449	30.8%
	**9**	**r-rho >0.1, r>0, rho <0**	**173**	0.14%	8	64	101	23.1%
	**10**	**r-rho >0.1, r>0, rho >0**	**11859**	9.35%	164	2257	9438	10.9%
		**Total**	126821	100%	16.1	43.5%	40.4%	

Note: GP-Gene pairs, P1-percentage of No. of gene pairs in total pairs, P2-percentage of the number of genes with normality in No. of GP. Shapiro-wilk testing with a cut-off values of 0.01. P-values <0.01 is considered non-normality, abs-absolute value.

The above classification of all gene pairs followed by Shapiro-Wilk test clearly indicates that the expression values of most genes do not obey a normal distribution, and that Pearson performed well even when a pair of genes' expression data are non-normal. For 84.46% gene pairs belong to the Category II (Table 4), all methods except the Distance Covariance (as shown in Figure 9) did not make much difference in identifying genes having normality distribution. For the rest 15.54% gene pairs of category I or III, all methods except the Pearson and Distance Covariance methods did not make significant difference in identifying genes with normality distribution when they were employed to analyze these pairs (Figure 9). Nevertheless, based on the patterns shown in Figure 9, the Weighted Rank method tended to identify more gene pairs with normal distribution.

## Discussion

We have shown the efficiencies of eight different gene association methods used to associate genes in a pairwise manner for pathway and network analysis. What is particularly important is that we showed all eight gene association methods can be plugged into the TF-Cluster package and lead to the discovery of genes controlling complex traits. This has significant implications in increasing crop and animal yield and quality in agricultural context and enhancing our understanding to the regulation of complex traits. Based on the principles of operation, the eight methods can be roughly classified into three categories: A) rank-based, including Spearman, Weighted Rank, Kendall, and Hoeffding, which use ranks of expression values instead of original values for analysis, and thus are robust to outliers, an observation that lies at an unusual distance from the rest of the data. B) regression based methods, including Theil-Sen and Rank Theil-Sen; C) dependence methods, which include Distance Covariance, and Pearson. From the analyses we have done, it is obvious that the performance of different methods is dependent on the principles of operation of each method, the properties of the data, and the biological events or biological processes in which genes have different behaviors. To unravel some underpinning mechanisms that are responsible for the performance discrepancy of different methods, we will discuss some factors that may play a role in gene association including the distribution of the data, ranking versus non-ranking, and the function of genes in particular clusters.

### Rank-based methods or non-rank-based methods?

Among the eight gene association methods, Spearman, Kendall, Weighted Rank, and Hoeffding, are nonparametric rank-based methods. This class of methods uses ranks for correlation and therefore provides a robust measure of a monotonic relationship between two continuous random variables. They are also useful with ordinal data and are generally more robust to outliers. For this reason, they are particularly suitable for identifying key genes that increase or decline in monotonic fashions in expression data collected during a biological process or developmental stage. In a previous study, the efficiency of the Kendall test and Spearman's rho test in detecting monotonic trends in time series data are compared [30] and the conclusion is that the two methods have similar powers that depend on the pre-assigned significance level, magnitude of trend, sample size, and the variation within a time series. That is, the bigger the absolute magnitude of trend, the more powerful is the test; as the sample size increases, the test becomes more powerful; and as the amount of variation increases within a time series, the power of the test decreases. When a trend is present, the power is also dependent on the distribution type and the skewed nature of the time series. However, Newson [30] has argued for the superiority of Kendall's *τ* over Spearman's correlation *rho* as a rank-based measure of correlation because confidence intervals for Spearman's *rho* are less reliable and less interpretable than confidence intervals for Kendall's *τ*-parameters. According to Fujita et al [16], the Hoeffding's *D* measure may be used to infer both nonlinear and non-monotonic relationships between gene expression profiles with full control of type I error. Theil-Sen and Rank Theil-Sen methods are regression-based methods. Theil-Sen estimator is a median of the slopes determined by all pairs of sample points, and it provides accurate estimate and confidence intervals even when the data are non-normal and heteroscedastic. Pearson's correlation is a measure of the linear relationship between two continuous random variables, and it assumes a bivariate normal distribution. Only when the sample size is large enough will the data be close to bivariate normal distribution. Nevertheless, Pearson correlation coefficient is highly informative about the degree of linear dependence between two random quantities regardless of whether their joint distribution is normal [31]. Pearson correlation coefficient provides an accurate and complete description of the association if the data are normal, and could have significant advantages for continuous data without obvious outliers [32]. Generally speaking, outliers can have great influence on Pearson's correlations but have no or very little influence on Rank-based methods [16], [33], [34]. Many outliers in applied settings reflect measurement failures or other factors to which the model is not intended to generalize. Univariate outliers do not exist with rank-based methods as data are converted to ranks. In this study, both *Arabidopsis* and human data were normalized with RMA algorithm, during which the original expression values were logged on the base of 2. After this normalization, the gene expression values generally vary from 3 to 14. In this circumstance, the effect of outliers has been significantly reduced. However, we still observed that methods that are robust to outliers performed significantly better. Note that Spearman method is a rank version of Pearson method. From the results shown in Tables 1 and 2 and Figures 3, 4, 5, 6, 7, a direct comparison of Spearman and Pearson methods showed Spearman performing better. Also Rank Theil-Sen method outperformed Theil-Sen method in most cases (Tables 1, 2, Figures 3, 4, 5, 6, 7).

Distance Covariance is a method that is analogous to product-moment covariance. It provides a natural extension of Pearson product-moment covariance for measuring dependence of bivariate variables in all types of applications [35]. Distance Covariance is sensitive to all types of departures from independence including nonlinear or non-monotonic dependence. In Monte Carlo studies, the Distance Covariance test exhibits superior power compared to relative to parametric or rank-based likelihood ratio tests again non-monotonic types of dependence. It has also been demonstrated that the test was quite competitive with the parametric likelihood ratio tests when applied to multivariate normal data. The practical message is that the Distance Covariance test is a powerful test for all types of dependence. In our study, we do find that Distance Covariance captured all kinds of relationships as evidenced by a longer coexpression gene list resulting from coexpression analysis, and a large cluster size resulting from triple-link algorithm. However, at high stringency, the discovery rate of positive genes of the Distance Covariance method is low. Although it captures all kinds of relationships, we currently cannot dissect these relationships into individual components [35]; otherwise, Distance Covariance would be the more useful method.

### Is normality a factor affecting the performance of different methods?

We found that all eight gene association methods tended to associate more genes with normality for Category II data as compared to either Category I or III data (Figure 9). On average, all methods except Pearson associated 11.8∼19.6% more genes with normality for Category II data than Category I data while Pearson associated only 4.8% more. All methods except Pearson and Distance Covariance associated 2.0∼4.7% more genes with normality for Category II data than Category III data while Pearson associated 15.7% more and Distance Covariance associated −1.9% less (Figure 9). However, further analysis of genes with normality in Category I, II, and II revealed that the baseline percentage of genes with normality in these three categories are 22.9%, 41.9% and 12.7% respectively (Table 4). It is obvious that all methods except Pearson tended to identify more genes with normal distribution in Category III than Category I though the baseline of genes with normal distribution in Category III is much low than that of Category I, indicating that the baselines in three categories can contribute differently to the percentage of the associated genes with normality, and that baseline is not the only factor that affects the percentage of the associated genes being normal distribution. Finally, the different rankings of eight gene associate methods in three categories indicate that there is an interactive effect between different method and data properties, which requires a specifically designed experiment to dissect. Based on the results shown in Figure 9, all methods except Pearson and Distance Covariance have only a relatively small discrepancy in identifying genes with normality with an occasional largest maximal difference of 6.8%.

### How to understand the different efficiencies of eight gene association methods in *Arabidopsis* and human?

Our study did not lead to the same rankings of the efficiencies of eight gene association methods from *Arabidopsis* and human data. In the pathway analysis, we showed different methods had distinct efficiencies when applied to different pathways in *Arabidopsis* (Figure 2). If this is the case, why we anticipate the consistent rankings to be obtained when these methods are used to different biological processes across two species? We would ascribe the disagreement in two species to the different biological processes we analyzed rather than the two species. It is conceivable that different biological processes take place in different scales (width), and complexity, and that underlying molecular regulatory mechanisms can be a single or multiple hierarchical modules in parallel, leading to coexpression occurring on a different scale. In addition, the regulatory networks of some biological processes involve self-regulatory, circuits, feedback loops, and feed forward mechanisms that make coordination of involved genes have different association strengths. Plants under stresses (*Arabidopsis* data) usually have wide-spectrum responses that are coordinated to help plants survive while human stem cells treated with reagents can disrupt pluripotency and induce differentiation known to have some regulatory circuit and feedback motifs [25], [36]. In addition, *Arabidopsis* roots harvested include more cell types than human stem cells that are relatively uniform, and the harvest time and time intervals can also affect gene association strength via gene profiles. All these aspects and unidentified hidden variables can lead to different TF behaviors, resulting in the different efficiency of the eight gene association methods in two species.

Although principles of statistical operation play a key role in determining the efficiency of different methods, we should not ignore the biological models underpinning each data set that can make a statistical method less efficient than another. An example for this is Pearson and Spearman methods. Charles Spearman proposed rank correlation in 1904 [37], a non-parametric version of the conventional Pearson correlation. However, his method was not appreciated by many colleagues mainly because the method appeared to have less power in statistics. We showed here that Spearman method has its applications in finding patterns from noisy gene expression data where more robust methods are demanded.

### How to identify the most appropriate method for studying biological processes of interest in a given data set?

Although we can opt for a method based on its principle of statistical operation without paying attention to the biological models in a given data set, this may not lead to a coordination network that will reveal biological knowledge. High dimensional biological data from microarray or high throughput sequencing data often contain at least a few hundred different biological processes. There is no statistical method that is suitable for all of them. Identification of the most efficient method for knowledge discovery of a specific biological process demands concrete pre-diagnostic analyses. Based on our study and our empirical knowledge, we would suggest the following procedure for identifying the most appropriate gene association method for a specific biological theme in a given data set: (1) Evaluate the prior knowledge of biological processes of one's interest, and select a few known genes involved in these processes; (2) Use the R codes from this study to perform a genome-wide coexpression analysis to obtain the top 100 or 500 genes that are most closely associated to the selected known genes; (3) Perform an evaluation of these 100 or 500 genes by examining which methods can associate the more functionally relevant genes to the selected genes. This can be achieved by examining gene annotation or performing GO term enrichment analysis: and (4) Choose the best method for the data. However, if prior knowledge of biological theme of one's interest is lacking, we suggest the most stable gene association method. Generally speaking, Spearman or Rank Theil-Sen is recommended for constructing co-expression network, and Hoeffding, Kendall or Spearman for pathway gene analysis.

## Conclusions

The analyses we have performed clearly demonstrate the distinct and common performance of eight gene association methods. For both pathway and network analyses, the Spearman, Kendall, Hoeffding, and Weighted Rank methods performed very well with some minor discrepancies, which are rooted in their similar principles of operation. The Rank Theil-Sen and Theil-Sen performed very well for network analysis but are not proficient in pathway analysis. The current challenge for implementing the Rank Theil-Sen and Theil-Sen lies in the much longer computational time. The Pearson and Distance Covariance methods are distinct and generally are less valuable for identifying biologically or functionally associated genes. Unfortunately, the efficiency of different methods indeed varies with the biological processes. For this reason, identification of the best method for a specific biological process requires some pre-analyses to be done first, which can be facilitated by the R programs we provided.

## Materials and Methods

### Pathway data

Pathway genes and annotation, AraCyc data, were obtained from TAIR (www.arabidopsis.org) as a flat file dump (aracyc_pathways.20110406) that listed accessions for 393 different pathways associated with 2101 unique genes. Most of AraCyc data was annotated based on experimental evidence while only a few were based on computational inference. All 9 pathways we analyzed were annotated based on experimental evidence (http://pmn.plantcyc.org). Affymetrix ATH1 GeneChip probe set and target gene information are from an annotations data file downloaded from the Affymetrix Web site in October, 2011.

### Microarray data sets

*Arabidopsis* compendium data were generated in 6 microarray experiments (GSE7636, 7639, 7641, 7642, 8787, 5623) in which *Arabidopsis* roots under salt stress conditions were harvested for RNA extraction and array hybridization. We downloaded data for each experiment from NCBI GEO website http://www.ncbi.nlm.nih.gov/geo and then pooled them together. All data mentioned above are derived from hybridization of Affymetrix 25 k ATH1 microarrays [38]. The original CEL files were processed by the robust multi-array analysis (RMA) algorithm [39] using the Bioconductor package. For quality control we used methods that were previously described [3]. This data set was recently used for identifying TFs involved in salt stress response and growth [1], [40].

The other data we used were generated from multiple microarray experiments of human stem cells at James Thomson's lab at University of Wisconsin at Madison. In each experiment, different reagents that disrupted pluripotency while triggering differentiation were used. We pooled the data of each experiment together and obtained a compendium data set containing 189 high-density human gene expression arrays, each with 36,398 human locus identifiers. The data set was normalized with RMA algorithm as described for *Arabidopsis* data [39] More detail of this data were described in our earlier publication [1].

### Kendall's rank correlation

Kendall's rank correlation is a non-parametric measure of the strength of the dependence between two variables. It measures the similarity of the ordering of the data when ranked by each of the variables.

Let 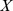 and 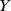 be the two random variables with observations 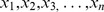 and 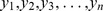 respectively. Any pair of observations 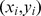 and 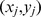 are said to be concordant if both 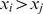 and 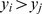 or if both 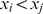 and 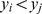 and they are said to be discordant if 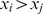
and 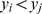 or if 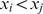 and. 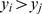 If 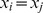 or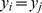, then the pair is neither concordant nor discordant.

Kendall's correlation coefficient is defined as [41]

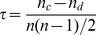
Where, 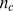  = number of concordant pairs. 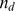  = number of discordant pairs. 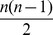  =  Total number of possible (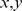) pairs.

If there are tied observations (the observations with same values), then the following formula is used to find the correlation coefficient
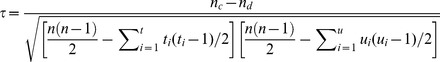
Where 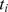 is the number of observations tied at a particular rank of 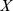 and 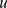 is the number of observations tied at a rank of 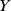. The value of the coefficient ranges from −1 to +1. If the ranks of the two variables are same, the value of the coefficient is 1 and if one ranking is reverse the other then the value is −1. If the two variables are independent, the value is approximately equal to zero.

Kendall's rank correlation coefficient provides a statistical test to test the independence of two variables. The test is non-parametric and does not make any assumption about the distributions of the variables.

Under the null hypothesis of 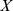 and 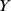 being independent, for a large sample, Kendall's correlation follows a normal distribution with mean 0 and variance 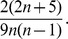 [20]. Therefore for large n, under null hypothesis, the statistic 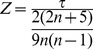 follows standard normal distribution. Kendall's correlation is robust to outliers. The R code for Kendall was adopted from R package stats (http://r-project.org).

### Pearson's correlation

Pearson's correlation coefficient measures the strength of the linear relationship between two random variables. The value of the correlation coefficient is between −1 and 1. The correlation closer to +1 or −1 indicates the relationship is closer to a perfect linear relationship. The two variables have positive association (the values of the one variable increases with the increase in the value of the other variable) if the value for correlation is positive and the variable have a negative association (the values of one variable decreases with the increase in the value of the other) if the value for the correlation is negative. If the two variables are uncorrelated, the Pearson's correlation is 0.

Suppose 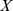 and 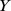 be two random variables with n measurements. Then the correlation between two variables is computed as
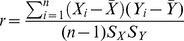
where, 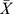 and 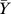 are the sample means and 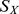 and 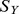 are the sample standard deviations of 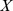 and 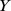
respectively.

Under the null hypothesis of two variables being independent, the quantity
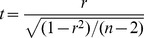
follows a Student's t distribution with n-2 degrees of freedom [42].

The Pearson's correlation assumes the data is normally distributed and there is a linear relationship between the two variables. It is sensitive to outliers and requires the data to be measured on interval or ratio scale. For R code see the cor.test package (http://r-project.org).

### Spearman's rank correlation

Spearman's rank correlation coefficient is a nonparametric measure of association. It assesses the nonlinear monotonic relationship between the two variables by the linear relationship between the ranks of the values of the two variables. Like other correlations, Spearman's correlation also takes values between −1 and +1. The positive correlation implies the ranks of both variables increase together and negative correlation implies the ranks of one variable increases as the ranks of the other variable decrease. A correlation close to zero means there is no linear relationship between the ranks of the two variables.

Spearman's correlation coefficient does not require the data to be measured on interval or ratio scale. It can be used for ordinal data. Spearman's correlation is computed the same way as the Pearson correlation but instead of using the original values of the variables, the ranks of the values are used [5]. The tied values are assigned a rank equal to the average of their positions in the ascending order of the values. In case of no tied ranks, the following formula can be used to find the correlation [43].
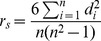
where; 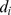 =  the difference between the ranks of the 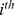 observations of the two variables. n =  the number of pairs of values.

Under the null hypothesis of statistical independence of the variables, for a sufficiently large sample the quantity


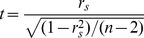
 follows a Student's t-distribution with n-2 degrees of freedom [44]. For R code see the cor.test package (http://r-project.org).

### Weighted rank correlation

The weighted rank correlation coefficient used in this study is proposed by Pinto da Costa and Soares [21]. It is adapted from Spearman's rank correlation but, unlike Spearman correlation coefficient, which treats all the ranks equally, the weighted rank correlation gives weight to the distance between two ranks using a linear function of those ranks. It gives more weight to higher ranks than the lower ranks.

Suppose 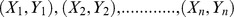 are the n paired observation of two random variable 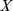 and 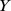 and let 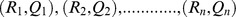 are the paired ranks of these observation. Then the weighted rank correlation of these two random variables is given by
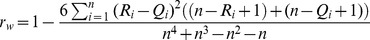



The values of 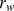 ranges from −1 to +1 and in case of 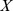 and 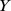 being independent, 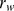 is 0. Under the hypothesis of independence between the two vectors of ranks, the expected value of 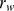 is 0 and variance of 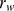 is. 
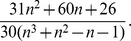
 The quantity 
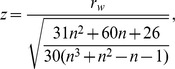
 follows a standard normal distribution. For more details please see [45].

### Distance covariance

Distance covariance provides a nonparametric test to test the statistical independence of two variables or vectors. Distance covariance and distance correlation are the measure of dependence between two random vectors of arbitrary dimensions [19]. The values of distance correlation range from 0 to 1 and distance covariance is greater than or equal to 0. The value of the distance covariance of two random variables is equal to 0 if and only if they are independent.

Suppose 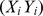, 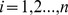 are pairs of measurements from two random variables 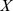 and 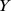. Let 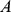 be a pairwise Euclidean distance matrix of 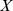 with 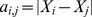 as the 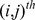 entry and 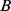 be a pairwise Euclidean distance matrix of 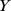 with as the 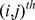 entry for 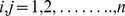, |.| denotes Euclidean norm. Then get the matrices 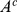 and 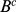 by centralizing the matrices 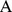 and 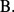


The 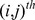 entry of 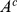 is 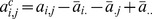 where 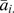 is the 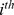 row mean, 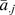 is the 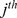 column mean, and 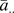 is the grand mean of 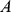. Similarly the 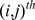 entry of 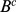 is 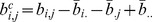 where 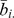 is the 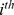 row mean, 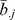 is the 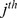 column mean, and 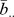 is the grand mean of 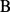. The squared distance covariance is the arithmetic average of the product of 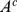 and 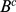, that is given as
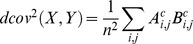



The statistic 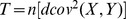 determines a consistent test of independence of random variables. The asymptotic distribution of 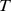 is a quadratic form centered Gaussian random variables, with coefficients depending on the distributions of 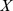
and 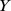. When the distributions of 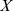 and 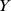 are unknown, the test based on 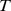 can be implemented as a permutation test. For more detail see [19], [35]. R code implementation was adopted from the *dcov.test* function in the *energy* package for R.

### Hoeffding’s measure of association

Hoeffding’s measure, 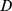, is a nonparametric measure of association. Considering the two random variables 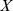 and 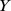 with continuous distribution functions, 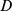 is defined as


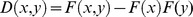
, where 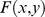 is the joint distribution of 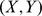 and 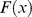, 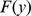 are the marginal distributions of 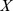 and 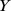 respectively. The random variables 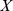 and 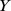 are independent if and only if 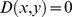. The statistic 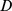 depends only on the ranks order of the observations and can be computed using the following formula [46]

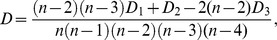
Where



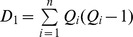


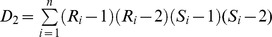


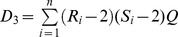




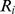
 is the rank of 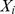, 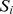 is the rank of 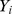 , and the bivariate rank, 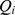, is the number of both 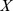 and 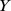 values less than the 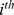 point and can be calculated as


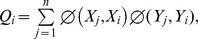
 where 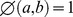 if 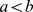 and 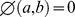 otherwise. So it gives the number of bivariate observations for which 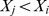 and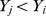.

### The test for independence

Given two random variables with continuous distribution functions, a test for independence can be carried out as follows:

At the significance level of 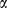, reject the null hypothesis of independence if and only if 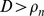, where 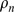 is given as


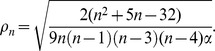
, and satisfies the inequality 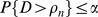 [12]

For more details please see [16], [46].

### Theil –Sen Estimator

Consider a simple linear regression model



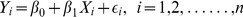
(1) to find the relationship between two variables 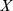 and 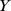. where 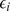 are independent and identically distributed random variables, and 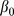 and 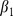 are unknown parameters. The slope 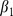 in equation 1 tells about the relationship between 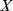 and 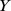. If the error term 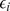 is normal, then the slope, 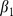, can be estimated with ordinary least square (OLS) estimator. But if 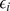 is non-normal and heteroscedastic, the ordinary least square estimator can be highly inefficient and the confidence interval s for the slope inaccurate. The Theil-Sen estimator of the slope proposed by Henri Theil and Pranab K. Sen provides an accurate estimate and confidence intervals even with non-normal data and heteroscedasticity. Theil-Sen estimator is a median of the slopes determined by all pairs of sample points. Considering two pairs of sample points 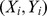 and 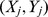. The slope determined by these points is 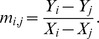 The Theil –Sen estimator of the slope, 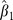, given as
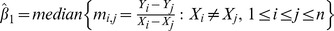
is a robust and unbiased estimator. It is less sensitive to outliers. It has a reasonably high break point of 29.3%, which means it can tolerate arbitrary corruption of up to 29.3% of the input data-points without degradation of its accuracy 18]. R code implementation is adopted from the *mblm* function in the *mblm* package for R.

### Rank Theil-Sen Estimator

In this study, we applied ranked observation to Theil-Sen estimator and named this approach Rank Theil-Sen Estimator.

## Supporting Information

Table S1 The selected pathways and pathway genes.
(XLS)

Table S2 Sensitivity, specificity, and predicted accuracy of eight gene association methods for pathway analysis.
(XLS)

Table S3 Positive gene lists for coexpression network construction and decomposition via TF-Cluster.
(PDF)

Table S4 Positive genes present in the major clusters shown in Figure 4 and Figure 6.
(XLS)

Doc S1 R-code for eight gene association methods.
(TXT)
