# Supplementary material for: Evaluation of Gene Association Methods for Coexpression Network Construction and Biological Knowledge Discovery
Source: PLoS One. 2012 Nov 30;7(11):e50411. doi: 10.1371/journal.pone.0050411 (PMC3511551; doi:10.1371/journal.pone.0050411)
Supplement: Table S3 — Positive gene lists for coexpression network construction and decomposition via TF-Cluster. (PDF) [file pone.0050411.s003.pdf]

Table S3. Positive gene lists for coexpression network construction and decomposition via TF-Cluster (Nie et al. *BMC Systems Biology* 2011, **5**:53)

Original Table 1: TF cluster identified with pluripotency of human embryonic stem cells

Genes	Symbol	Description	Evidence
Cluster 1: A cluster of TFs control pluripotency renewal of human stem cells			
NM_024865	NANOG	Nanog homeobox	(BOYER *et al.* 2005)
BC099704	NANOGP8	Nanog homeobox pseudogene 8	Pseudogene with similarity to Nanog.
NM_003106	SOX2	SRY box 2	(BOYER *et al.* 2005)
NM_002701	POU5F1	POU class 5 homeobox 1	(BOYER *et al.* 2005)
NM_006892	DNMT3B	DNS methyltransferase 3 beta	(AASEN *et al.* 2008; GOPALAKRISHNAN *et al.* 2009)
NM_080618	CTCFL	CCCTC-binding factor-like	(MONK *et al.* 2008)
NM_016089	ZNF589	Zinc finger 589	(GIORGETTI *et al.* 2009; GREBER *et al.* 2007)
NM_004426	PHC1	Polyhomeotic homolog 1	(GIULIANO *et al.* 2005)
NM_005407	SALL2	SAL2 like	(GIORGETTI *et al.* 2009)
NM_004427	PHC2	Polyhomeotic homolog 2	(ISONO *et al.* 2005)
NM_032805	ZFP206	Zinc finger protein 206 (ZSCAN10)	(WANG *et al.* 2007; YU *et al.* 2009)
NM_003325	HIRA	HIR Histone Cell Cycle regulator	(ZWAKA 2006)
BC098403	ETV1	ETS variant 1	(FOUSE *et al.* 2008; LOH *et al.* 2006)
NM_006079	CITED2	Cbp/p300-interacting transactivator	(KRANC *et al.* 2003; KRANC *et al.* 2009)
NM_006074	TRIM22	Tripartite motif-containing 22	(GREBER *et al.* 2007)
XM_929986	LOC653441	Similar to polyhomeotic 1-like	Gene with sequence similarity to PHC1
Cluster 22: A cluster of TFs control neural development in earlier differentiation of human stem cells			
BC008687	NEUROG1	Neurogenin 1	(HIRABAYASHI *et al.* 2004)
NM_006161	NEUROG1	Neurogenin 1	(HIRABAYASHI *et al.* 2004)
NM_033178	DUX4	Double homeobox 4	(BOSNAKOVSKI *et al.* 2009)
NM_006732	FOSB	FBJ oncogene homolog B	(KELZ and NESTLER 2000)
NM_003317	TITF1	NK2 homeobox 1	(GARCIA-BARCELO *et al.* 2007)
NM_002478	MYOD1	myogenic differentiation 1	(DAUBAS *et al.* 2009; DAUBAS *et al.* 2000)
NM_006192	PAX1	Paired box 1	(JOOSTEN *et al.* 2005)
NM_002700	POU4F3	POU class 4 homeobox 3	(VAHAVA *et al.* 1998)
BC10493	POU4F3	POU class 4 homeobox 3	(VAHAVA *et al.* 1998)
Cluster 17: A cluster of TFs control differentiation towards multiple directions in human stem cells			
NM_001002295	GATA3	GATA binding protein 3	Trophectoderm (HOME *et al.* 2009)
NM_012258	HEY1	Hairy/enhancer-of-split related with YRPW motif 1	Trophectoderm (XU 2006)
NM_032638	GATA2	GATA binding protein 2	Trophectoderm (MA *et al.* 1997)
NM_030379	GLI2	GLI family zinc finger 2	Mesoderm(BUTTITTA *et al.* 2003)
NM_017410	HOXC13	Homeobox C13	Ectoderm(GODWIN and CAPECCHI 1999)
NM_002202	ISL1	ISL LIM homeobox 1	Mesoderm (CAI *et al.* 2003)
NM_005461	MAFB	v-maf musculoaponeurotic fibrosarcoma oncogene homolog B (avian)	Neural (STURGEON *et al.*)
NM_002449	MSX2	Msh homeobox 2	Mesoderm(BRUNELLI and COSSU 2005)
NM_175747	OLIG3	Oligodendrocyte transcription factor 3	Neural (FILIPPI *et al.* 2005)
NM_006099	PIAS3	Protein inhibitor of activated STAT, 3	Neural (ONISHI *et al.* 2009)
NM_019854	PRMT8	Protein arginine methyltransferase 8	Neural (LEE *et al.* 2005)
NM_030567	PRR7	Proline rich 7 (synaptic)	(MURATA *et al.* 2005)
NM_003068	SNAI2	Snail homolog 2 (Drosophila)	Neural Crest(STEGMANN *et al.* 1999)
NM_031439	SOX7	SRY (sex determining region Y)-box 7	Endoderm (Parietal) (FUTAKI *et al.* 2004)
NM_007129	ZIC2	Zic family member 2 (odd-paired homolog, Drosophila)	Neural (ELMS *et al.* 2003)

Original Table 2. Cluster 1, 2, 5, 7 and 19 identified from salt stress data of Arabidopsis roots containing rootgrowth and development (Nie et al 2011)

**Gene**	**Symbol**	**Description**	**Evidence**
Cluster 1: A TF cluster controlling the root hair growth			
AT5G58010	LRL3	Roothairless1	(KARAS *et al.* 2009)
AT5G19790	RAP2.11	Ethylene response factor controlling root growth	(JUNG *et al.* 2007)
AT1G27740	RSL4	Postmitotic cell growth in root-hair cells	(YI *et al.* 2010)
AT1G66470	RHD6	Early root hair formation	(MENAND *et al.* 2007; SINGH *et al.* 2008)
AT5G25810	TINY	ERF/AP2 TF control cell expansion in root	(WILSON *et al.* 1996)
AT2G28160	FRU	Regulates iron uptake responses in outer cells of root	(JAKOBY *et al.* 2004; YUAN *et al.* 2005)
Cluster 2: A cluster of TFs control root cap development (stem cells of roots)			
AT1G33280	BRN1	BRN1, SMB control root cap maturation	(BENNETT *et al.* 2010)
AT4G10350	BRN2	BRN2, SMB control root cap maturation	(BENNETT *et al.* 2010)
AT1G79580	SMB	FEZ and SMB control root stem cells	(WILLEMSEN *et al.* 2008)
AT5G39820	ANAC094	Apical meristem protein, function unknown	(YI *et al.* 2010)
AT1G26870	FEZ	FEZ and SMB control root stem cells in cap	(WILLEMSEN *et al.* 2008)
AT1G74500	TOM7	Embryonic root initiation	(SCHLERETH *et al.* 2010)
AT3G27010	TCP20	Postembryonic cell division in root	(LI *et al.* 2005)
AT2G30340	LBD13	Expressed in cells at the adaxial base of lateral roots	(SHUAI *et al.* 2002)
AT2G40470	LBD15	Expressed in cells at the adaxial base of lateral roots	(SHUAI *et al.* 2002)
AT1G51190	PLT2	Control root stem cell activity near cap	(ZHOU *et al.* 2010)
AT1G66350	RGL1	Root epidermal differentiation	(GAN *et al.* 2007)
AT2G37260	TTG2	Differentiation of trichomes and root hairless cells	(ISHIDA *et al.* 2007)
AT5G57420	IAA33	IAA is involved in root development	(KARES *et al.* 1990; PETERSSON *et al.* 2009)
Cluster 5: A cluster of TFs control root vascular development, second wall growth development			
AT1G71930	VND7	Regulates xylem vessel formation	(KUBO *et al.* 2005)
AT5G12870	MYB46	Target of SND1, control second wall biosynthesis	(ZHONG *et al.* 2007)
AT1G01780	LIM	LIM domain-containing protein	
AT1G12260	VND4	Switches for protoxylem and metaxylem vessel formation	(KUBO *et al.* 2005)
AT1G17950	MYB52	Second wall growth	(ZHONG *et al.* 2008)
AT1G63910	MYB103	Second wall growth	(ZHONG *et al.* 2008)
AT1G66230	MYB20	Second wall growth	(ZHONG *et al.* 2008)
AT1G68810	bHLH	Root vascular initial	(OHASHI-ITO and BERGMANN 2007)
AT1G73410	MYB54	Second wall growth	(ZHONG *et al.* 2008)
AT2G39830	DAR2	DA-1 related, control organ size	(LI *et al.* 2008)
AT2G45420	LBD18	Lateral root and tracheary element formation	(LEE *et al.* 2009)
AT3G21270	ADOF2	Early stages of vascular development	(GARDINER *et al.* 2010)
AT4G00220	JLO	A central regulator of auxin distribution and signaling in root	(BUREAU *et al.* 2010)
AT4G28500	SND2	Vascular cell differentiation	(GRANT *et al.* 2010)
AT5G66610	DAR7	DA-1 related, control organ size	(LI *et al.* 2008)
Cluster 7: A cluster of TFs that controlling root cell cycle & growth			
AT5G24330	AtXR6	Cell cycle regulation of late G1 to S phase	(RAYNAUD *et al.* 2006)
AT3G01330	DEL3	Cyclin D/retinoblastoma/E2F pathway	(SOZZANI *et al.* 2009)
AT2G22840	AtGRF1	Growth factor expressed in root	(KIM *et al.* 2003; KIM and KENDE 2004)
AT2G36400	AtGRF3	Growth factor expressed in root	(KIM *et al.* 2003; KIM and KENDE 2004)
AT4G37740	AtGRF2	Growth factor expressed in root	(KIM *et al.* 2003; KIM and KENDE 2004)
AT3G50870	MNP	GATA transcription factor	(OHASHI 2006)
AT1G34355	PS1	Parallel spindle 1 involved in meiosis	(D'ERFURTH *et al.* 2008)
AT4G23800	HMG1/HMG2	High mobile group 1, 2	(MENGES *et al.* 2002)
Cluster 19: A cluster of TFs control drought stress in response to ABA			
AT2G46270	GBF3	induced by ABA under water deprivation	(SEHNKE *et al.* 2005)
AT3G19290	ABF4	Regulate ABRE-dependent ABA signaling involved in drought stress	(YOSHIDA *et al.* 2010)
AT1G51140	bHLH	Drought stress	(RIZHSKY *et al.* 2004)
AT1G52890	ANAC019	Bind to drought-responsive cis-element in response to ABA	(JIANG *et al.* 2009; TRAN *et al.* 2004)
AT1G73730	EIL3	Ethylene signaling	(BINDER *et al.* 2007)
AT2G46680	ATHB7	Growth regulator in response to ABA	(OLSSON *et al.* 2004)

AT3G61890	ATHB12	Growth regulator in response to ABA	(OLSSON *et al.* 2004)
AT4G21440	MYB102	ABA-induced protein	(LEONHARDT *et al.* 2004)
AT4G25480	DREB1A	Drought stress genes responsive to ABA	(RIECHMANN *et al.* 2000) (LIU *et al.* 1998)
AT4G27410	RD26	Transcriptional activator in ABA-mediated dehydration response	(TRAN *et al.* 2004)
AT4G34000	ABF3	Regulate ABRE-dependent ABA signaling involved in drought stress	(Yoshida et al. 2010)
AT5G47640	NF-YB2	NF-YB2 (NUCLEAR FACTOR Y, SUBUNIT B2); transcription factor	(KUMIMOTO *et al.* 2011)
AT2G18550	HB-2	DNA binding / transcription factor	(SON *et al.* 2005)
AT1G77200	DREBA4	DREB subfamily A-4 of ERF/AP2 transcription factor	(RIECHMANN *et al.* 2000)

AASEN, T., A. RAYA, M. J. BARRERO, E. GARRETA, A. CONSIGLIO *et al.*, 2008 Efficient and rapid generation of induced pluripotent stem cells from human keratinocytes. Nat Biotechnol **26:** 1276-1284.

BENNETT, T., A. VAN DEN TOORN, G. F. SANCHEZ-PEREZ, A. CAMPILHO, V. WILLEMSEN *et al.*, 2010 SOMBRERO, BEARSKIN1, and BEARSKIN2 regulate root cap maturation in Arabidopsis. Plant Cell **22:** 640-654.

BINDER, B. M., J. M. WALKER, J. M. GAGNE, T. J. EMBORG, G. HEMMANN *et al.*, 2007 The Arabidopsis EIN3 binding F-Box proteins EBF1 and EBF2 have distinct but overlapping roles in ethylene signaling. Plant Cell **19:** 509-523.

BOSNAKOVSKI, D., R. S. DAUGHTERS, Z. XU, J. M. SLACK and M. KYBA, 2009 Biphasic myopathic phenotype of mouse DUX, an ORF within conserved FSHD-related repeats. PLoS One **4:** e7003.

BOYER, L. A., T. I. LEE, M. F. COLE, S. E. JOHNSTONE, S. S. LEVINE *et al.*, 2005 Core transcriptional regulatory circuitry in human embryonic stem cells. Cell **122:** 947-956.

BRUNELLI, S., and G. COSSU, 2005 A role for MSX2 and necdin in smooth muscle differentiation of mesoangioblasts and other mesoderm progenitor cells. Trends Cardiovasc Med **15:** 96-100.

BUREAU, M., M. I. RAST, J. ILLMER and R. SIMON, 2010 JAGGED LATERAL ORGAN (JLO) controls auxin dependent patterning during development of the Arabidopsis embryo and root. Plant Mol Biol **74:** 479-491.

BUTTITTA, L., R. MO, C. C. HUI and C. M. FAN, 2003 Interplays of Gli2 and Gli3 and their requirement in mediating Shh-dependent sclerotome induction. Development **130:** 6233-6243.

CAI, C. L., X. LIANG, Y. SHI, P. H. CHU, S. L. PFAFF *et al.*, 2003 Isl1 identifies a cardiac progenitor population that proliferates prior to differentiation and contributes a majority of cells to the heart. Dev Cell **5:** 877-889.

D'ERFURTH, I., S. JOLIVET, N. FROGER, O. CATRICE, M. NOVATCHKOVA *et al.*, 2008 Mutations in AtPS1 (Arabidopsis thaliana parallel spindle 1) lead to the production of diploid pollen grains. PLoS Genet **4:** e1000274.

DAUBAS, P., C. G. CRIST, L. BAJARD, F. RELAIX, E. PECNARD *et al.*, 2009 The regulatory mechanisms that underlie inappropriate transcription of the myogenic determination gene Myf5 in the central nervous system. Dev Biol **327:** 71-82.

DAUBAS, P., S. TAJBAKHSH, J. HADCHOUEL, M. PRIMIG and M. BUCKINGHAM, 2000 Myf5 is a novel early axonal marker in the mouse brain and is subjected to post-transcriptional regulation in neurons. Development **127:** 319-331.

ELMS, P., P. SIGGERS, D. NAPPER, A. GREENFIELD and R. ARKELL, 2003 Zic2 is required for neural crest formation and hindbrain patterning during mouse development. Dev Biol **264:** 391-406.

FILIPPI, A., N. TISO, G. DEFLORIAN, E. ZECCHIN, M. BORTOLUSSI *et al.*, 2005 The basic helix-loop-helix olig3 establishes the neural plate boundary of the trunk and is necessary for development of the dorsal spinal cord. Proc Natl Acad Sci U S A **102:** 4377-4382.

FOUSE, S. D., Y. SHEN, M. PELLEGRINI, S. COLE, A. MEISSNER *et al.*, 2008 Promoter CpG methylation contributes to ES cell gene regulation in parallel with Oct4/Nanog, PcG complex, and histone H3 K4/K27 trimethylation. Cell Stem Cell **2:** 160-169.

FUTAKI, S., Y. HAYASHI, T. EMOTO, C. N. WEBER and K. SEKIGUCHI, 2004 Sox7 plays crucial roles in parietal endoderm differentiation in F9 embryonal carcinoma cells through regulating Gata-4 and Gata-6 expression. Mol Cell Biol **24:** 10492-10503.

GAN, Y., H. YU, J. PENG and P. BROUN, 2007 Genetic and molecular regulation by DELLA proteins of trichome development in Arabidopsis. Plant Physiol **145:** 1031-1042.

GARCIA-BARCELO, M. M., D. K. LAU, E. S. NGAN, T. Y. LEON, T. T. LIU *et al.*, 2007 Evaluation of the thyroid transcription factor-1 gene (TITF1) as a Hirschsprung's disease locus. Ann Hum Genet **71:** 746-754.

GARDINER, J., I. SHERR and E. SCARPELLA, 2010 Expression of DOF genes identifies early stages of vascular development in Arabidopsis leaves. Int J Dev Biol **54:** 1389-1396.

GIORGETTI, A., N. MONTSERRAT, T. AASEN, F. GONZALEZ, I. RODRIGUEZ-PIZA *et al.*, 2009 Generation of induced pluripotent stem cells from human cord blood using OCT4 and SOX2. Cell Stem Cell **5:** 353-357.

GIULIANO, C. J., J. S. KERLEY-HAMILTON, T. BEE, S. J. FREEMANTLE, R. MANICKARATNAM *et al.*, 2005 Retinoic acid represses a cassette of candidate pluripotency chromosome 12p genes during induced loss of human embryonal carcinoma tumorigenicity. Biochim Biophys Acta **1731:** 48-56.

GODWIN, A. R., and M. R. CAPECCHI, 1999 Hair defects in Hoxc13 mutant mice. J Investig Dermatol Symp Proc **4:** 244-247.

GOPALAKRISHNAN, S., B. O. VAN EMBURGH, J. SHAN, Z. SU, C. R. FIELDS *et al.*, 2009 A novel DNMT3B splice variant expressed in tumor and pluripotent cells modulates genomic DNA methylation patterns and displays altered DNA binding. Mol Cancer Res **7:** 1622-1634.

GRANT, E. H., T. FUJINO, E. P. BEERS and A. M. BRUNNER, 2010 Characterization of NAC domain transcription factors implicated in control of vascular cell differentiation in Arabidopsis and Populus. Planta **232:** 337-352.

GREBER, B., H. LEHRACH and J. ADJAYE, 2007 Silencing of core transcription factors in human EC cells highlights the importance of autocrine FGF signaling for self-renewal. BMC Dev Biol **7:** 46.

HIRABAYASHI, Y., Y. ITOH, H. TABATA, K. NAKAJIMA, T. AKIYAMA *et al.*, 2004 The Wnt/beta-catenin pathway directs neuronal differentiation of cortical neural precursor cells. Development **131:** 2791-2801.

HOME, P., S. RAY, D. DUTTA, I. BRONSHTEYN, M. LARSON *et al.*, 2009 GATA3 is selectively expressed in the trophectoderm of peri-implantation embryo and directly regulates Cdx2 gene expression. J Biol Chem **284:** 28729-28737.

ISHIDA, T., S. HATTORI, R. SANO, K. INOUE, Y. SHIRANO *et al.*, 2007 Arabidopsis TRANSPARENT TESTA GLABRA2 is directly regulated by R2R3 MYB transcription factors and is involved in regulation of GLABRA2 transcription in epidermal differentiation. Plant Cell **19:** 2531-2543.

ISONO, K., Y. FUJIMURA, J. SHINGA, M. YAMAKI, O. W. J *et al.*, 2005 Mammalian polyhomeotic homologues Phc2 and Phc1 act in synergy to mediate polycomb repression of Hox genes. Mol Cell Biol **25:** 6694-6706.

JAKOBY, M., H. Y. WANG, W. REIDT, B. WEISSHAAR and P. BAUER, 2004 FRU (BHLH029) is required for induction of iron mobilization genes in Arabidopsis thaliana. FEBS Lett **577:** 528-534.

JIANG, H., H. LI, Q. BU and C. LI, 2009 The RHA2a-interacting proteins ANAC019 and ANAC055 may play a dual role in regulating ABA response and jasmonate response. Plant Signal Behav **4:** 464-466.

JOOSTEN, P. H., E. J. VAN ZOELEN and C. MURRE, 2005 Pax1/E2a double-mutant mice develop non-lethal neural tube defects that resemble human malformations. Transgenic Res **14:** 983-987.

JUNG, J., S. Y. WON, S. C. SUH, H. KIM, R. WING *et al.*, 2007 The barley ERF-type transcription factor HvRAF confers enhanced pathogen resistance and salt tolerance in Arabidopsis. Planta **225:** 575-588.

KARAS, B., L. AMYOT, C. JOHANSEN, S. SATO, S. TABATA *et al.*, 2009 Conservation of lotus and Arabidopsis basic helix-loop-helix proteins reveals new players in root hair development. Plant Physiol **151:** 1175-1185.

KARES, C., E. PRINSEN, H. VAN ONCKELEN and L. OTTEN, 1990 IAA synthesis and root induction with iaa genes under heat shock promoter control. Plant Mol Biol **15:** 225-236.

KELZ, M. B., and E. J. NESTLER, 2000 deltaFosB: a molecular switch underlying long-term neural plasticity. Curr Opin Neurol **13:** 715-720.

KIM, J. H., D. CHOI and H. KENDE, 2003 The AtGRF family of putative transcription factors is involved in leaf and cotyledon growth in Arabidopsis. Plant J **36:** 94-104.

KIM, J. H., and H. KENDE, 2004 A transcriptional coactivator, AtGIF1, is involved in regulating leaf growth and morphology in Arabidopsis. Proc Natl Acad Sci U S A **101:** 13374-13379.

KRANC, K. R., S. D. BAMFORTH, J. BRAGANCA, C. NORBURY, M. VAN LOHUIZEN *et al.*, 2003 Transcriptional coactivator Cited2 induces Bmi1 and Mel18 and controls fibroblast proliferation via Ink4a/ARF. Mol Cell Biol **23:** 7658-7666.

KRANC, K. R., H. SCHEPERS, N. P. RODRIGUES, S. BAMFORTH, E. VILLADSEN *et al.*, 2009 Cited2 is an essential regulator of adult hematopoietic stem cells. Cell Stem Cell **5:** 659-665.

KUBO, M., M. UDAGAWA, N. NISHIKUBO, G. HORIGUCHI, M. YAMAGUCHI *et al.*, 2005 Transcription switches for protoxylem and metaxylem vessel formation. Genes Dev **19:** 1855-1860.

KUMIMOTO, R., C. SIRIWARDANA, K. GAYLER and J. H. I. RISINGER, B., 2011 The Roles of NF-Y Transcription Factors in ABA Responses. 22nd Internaltional Conference on Arabidopsis Research.

LEE, H. W., N. Y. KIM, D. J. LEE and J. KIM, 2009 LBD18/ASL20 regulates lateral root formation in combination with LBD16/ASL18 downstream of ARF7 and ARF19 in Arabidopsis. Plant Physiol **151:** 1377-1389.

LEE, J., J. SAYEGH, J. DANIEL, S. CLARKE and M. T. BEDFORD, 2005 PRMT8, a new membrane-bound tissue-specific member of the protein arginine methyltransferase family. J Biol Chem **280:** 32890-32896.

LEONHARDT, N., J. M. KWAK, N. ROBERT, D. WANER, G. LEONHARDT *et al.*, 2004 Microarray expression analyses of Arabidopsis guard cells and isolation of a recessive abscisic acid hypersensitive protein phosphatase 2C mutant. Plant Cell **16:** 596-615.

LI, C., T. POTUSCHAK, A. COLON-CARMONA, R. A. GUTIERREZ and P. DOERNER, 2005 Arabidopsis TCP20 links regulation of growth and cell division control pathways. Proc Natl Acad Sci U S A **102:** 12978-12983.

LI, Y., L. ZHENG, F. CORKE, C. SMITH and M. W. BEVAN, 2008 Control of final seed and organ size by the DA1 gene family in Arabidopsis thaliana. Genes Dev **22:** 1331-1336.

LIU, Q., M. KASUGA, Y. SAKUMA, H. ABE, S. MIURA *et al.*, 1998 Two transcription factors, DREB1 and DREB2, with an EREBP/AP2 DNA binding domain separate two cellular signal transduction pathways in drought- and low-temperature-responsive gene expression, respectively, in Arabidopsis. Plant Cell **10:** 1391-1406.

LOH, Y. H., Q. WU, J. L. CHEW, V. B. VEGA, W. ZHANG *et al.*, 2006 The Oct4 and Nanog transcription network regulates pluripotency in mouse embryonic stem cells. Nat Genet **38:** 431-440.

MA, G. T., M. E. ROTH, J. C. GROSKOPF, F. Y. TSAI, S. H. ORKIN *et al.*, 1997 GATA-2 and GATA-3 regulate trophoblast-specific gene expression in vivo. Development **124:** 907-914.

MENAND, B., K. YI, S. JOUANNIC, L. HOFFMANN, E. RYAN *et al.*, 2007 An ancient mechanism controls the development of cells with a rooting function in land plants. Science **316:** 1477-1480.

MENGES, M., L. HENNIG, W. GRUISSEM and J. A. MURRAY, 2002 Cell cycle-regulated gene expression in Arabidopsis. J Biol Chem **277:** 41987-42002.

MONK, M., M. HITCHINS and S. HAWES, 2008 Differential expression of the embryo/cancer gene ECSA(DPPA2), the cancer/testis gene BORIS and the pluripotency structural gene OCT4, in human preimplantation development. Mol Hum Reprod **14:** 347-355.

MURATA, Y., T. DOI, H. TANIGUCHI and Y. FUJIYOSHI, 2005 Proteomic analysis revealed a novel synaptic proline-rich membrane protein (PRR7) associated with PSD-95 and NMDA receptor. Biochem Biophys Res Commun **327:** 183-191.

OHASHI, K., 2006 [HANABA TARANU, a GATA transcription factor which affects shoot apical meristem development]. Seikagaku **78:** 888-891.

OHASHI-ITO, K., and D. C. BERGMANN, 2007 Regulation of the Arabidopsis root vascular initial population by LONESOME HIGHWAY. Development **134:** 2959-2968.

OLSSON, A. S., P. ENGSTROM and E. SODERMAN, 2004 The homeobox genes ATHB12 and ATHB7 encode potential regulators of growth in response to water deficit in Arabidopsis. Plant Mol Biol **55:** 663-677.

ONISHI, A., G. H. PENG, C. HSU, U. ALEXIS, S. CHEN *et al.*, 2009 Pias3-dependent SUMOylation directs rod photoreceptor development. Neuron **61:** 234-246.

PETERSSON, S. V., A. I. JOHANSSON, M. KOWALCZYK, A. MAKOVEYCHUK, J. Y. WANG *et al.*, 2009 An auxin gradient and maximum in the Arabidopsis root apex shown by high-resolution cell-specific analysis of IAA distribution and synthesis. Plant Cell **21:** 1659-1668.

RAYNAUD, C., R. SOZZANI, N. GLAB, S. DOMENICHINI, C. PERENNES *et al.*, 2006 Two cell-cycle regulated SET-domain proteins interact with proliferating cell nuclear antigen (PCNA) in Arabidopsis. Plant J **47:** 395-407.

RIECHMANN, J. L., J. HEARD, G. MARTIN, L. REUBER, C. JIANG *et al.*, 2000 Arabidopsis transcription factors: genome-wide comparative analysis among eukaryotes. Science **290:** 2105-2110.

RIZHSKY, L., H. LIANG, J. SHUMAN, V. SHULAEV, S. DAVLETOVA *et al.*, 2004 When defense pathways collide. The response of Arabidopsis to a combination of drought and heat stress. Plant Physiol **134:** 1683-1696.

SCHLERETH, A., B. MOLLER, W. LIU, M. KIENTZ, J. FLIPSE *et al.*, 2010 MONOPTEROS controls embryonic root initiation by regulating a mobile transcription factor. Nature **464:** 913-916.

SEHNKE, P. C., B. J. LAUGHNER, C. R. LYERLY LINEBARGER, W. B. GURLEY and R. J. FERL, 2005 Identification and characterization of GIP1, an Arabidopsis thaliana protein that enhances the DNA binding affinity and reduces the oligomeric state of G-box binding factors. Cell Res **15:** 567-575.

SHUAI, B., C. G. REYNAGA-PENA and P. S. SPRINGER, 2002 The lateral organ boundaries gene defines a novel, plant-specific gene family. Plant Physiol **129:** 747-761.

SINGH, S. K., U. FISCHER, M. SINGH, M. GREBE and A. MARCHANT, 2008 Insight into the early steps of root hair formation revealed by the procuste1 cellulose synthase mutant of Arabidopsis thaliana. BMC Plant Biol **8:** 57.

SON, O., H. Y. CHO, M. R. KIM, H. LEE, M. S. LEE *et al.*, 2005 Induction of a homeodomain-leucine zipper gene by auxin is inhibited by cytokinin in Arabidopsis roots. Biochem Biophys Res Commun **326:** 203-209.

SOZZANI, R., C. MAGGIO, R. GIORDO, E. UMANA, J. T. ASCENCIO-IBAÑEZ *et al.*, 2009 The E2FD/DEL2 factor is a component of a regulatory network controlling cell proliferation and development in Arabidopsis. Plant Molecular Biology **72:** 381-395.

STEGMANN, K., J. BOECKER, C. KOSAN, A. ERMERT, J. KUNZ *et al.*, 1999 Human transcription factor SLUG: mutation analysis in patients with neural tube defects and identification of a missense mutation (D119E) in the Slug subfamily-defining region. Mutat Res **406:** 63-69.

STURGEON, K., T. KANEKO, M. BIEMANN, A. GAUTHIER, K. CHAWENGSAKSOPHAK *et al.*, Cdx1 refines positional identity of the vertebrate hindbrain by directly repressing Mafb expression. Development **138:** 65-74.

TRAN, L. S., K. NAKASHIMA, Y. SAKUMA, S. D. SIMPSON, Y. FUJITA *et al.*, 2004 Isolation and functional analysis of Arabidopsis stress-inducible NAC transcription factors that bind to a drought-responsive cis-element in the early responsive to dehydration stress 1 promoter. Plant Cell **16:** 2481-2498.

VAHAVA, O., R. MORELL, E. D. LYNCH, S. WEISS, M. E. KAGAN *et al.*, 1998 Mutation in transcription factor POU4F3 associated with inherited progressive hearing loss in humans. Science **279:** 1950-1954.

WANG, Z. X., J. L. KUEH, C. H. TEH, M. ROSSBACH, L. LIM *et al.*, 2007 Zfp206 is a transcription factor that controls pluripotency of embryonic stem cells. Stem Cells **25:** 2173-2182.

WILLEMSEN, V., M. BAUCH, T. BENNETT, A. CAMPILHO, H. WOLKENFELT *et al.*, 2008 The NAC domain transcription factors FEZ and SOMBRERO control the orientation of cell division plane in Arabidopsis root stem cells. Dev Cell **15:** 913-922.

WILSON, K., D. LONG, J. SWINBURNE and G. COUPLAND, 1996 A Dissociation insertion causes a semidominant mutation that increases expression of TINY, an Arabidopsis gene related to APETALA2. Plant Cell **8:** 659-671.

XU, R. H., 2006 In vitro induction of trophoblast from human embryonic stem cells. Methods Mol Med **121:** 189-202.

YI, K., B. MENAND, E. BELL and L. DOLAN, 2010 A basic helix-loop-helix transcription factor controls cell growth and size in root hairs. Nat Genet **42:** 264-267.

YOSHIDA, T., Y. FUJITA, H. SAYAMA, S. KIDOKORO, K. MARUYAMA *et al.*, 2010 AREB1, AREB2, and ABF3 are master transcription factors that cooperatively regulate ABRE-dependent ABA

signaling involved in drought stress tolerance and require ABA for full activation. Plant J **61:** 672-685.

YU, H. B., G. KUNARSO, F. H. HONG and L. W. STANTON, 2009 Zfp206, Oct4, and Sox2 are integrated components of a transcriptional regulatory network in embryonic stem cells. J Biol Chem **284:** 31327-31335.

YUAN, Y. X., J. ZHANG, D. W. WANG and H. Q. LING, 2005 AtbHLH29 of Arabidopsis thaliana is a functional ortholog of tomato FER involved in controlling iron acquisition in strategy I plants. Cell Res **15:** 613-621.

ZHONG, R., C. LEE, J. ZHOU, R. L. MCCARTHY and Z. H. YE, 2008 A battery of transcription factors involved in the regulation of secondary cell wall biosynthesis in Arabidopsis. Plant Cell **20:** 2763-2782.

ZHONG, R., E. A. RICHARDSON and Z. H. YE, 2007 The MYB46 transcription factor is a direct target of SND1 and regulates secondary wall biosynthesis in Arabidopsis. Plant Cell **19:** 2776-2792.

ZHOU, W., L. WEI, J. XU, Q. ZHAI, H. JIANG *et al.*, 2010 Arabidopsis Tyrosylprotein sulfotransferase acts in the auxin/PLETHORA pathway in regulating postembryonic maintenance of the root stem cell niche. Plant Cell **22:** 3692-3709.

ZWAKA, T. P., 2006 Breathing chromatin in pluripotent stem cells. Dev Cell **10:** 1-2.
